# Order–Disorder Balance in Silk-Elastin-like Polypeptides Determines Their Self-Assembly into Hydrogel Networks

**DOI:** 10.1021/acsami.4c17903

**Published:** 2024-12-16

**Authors:** Diego López Barreiro, Klaartje Houben, Olaf Schouten, Gijsje H. Koenderink, Jens C. Thies, Cees M. J. Sagt

**Affiliations:** 1 Manufacturing Futures Lab, Department of Chemical Engineering, University College London, London WC1E 7JE, United Kingdom; 2 Centre for Nature-Inspired Engineering, Department of Chemical Engineering, University College London, London WC1E 7JE, United Kingdom; 3 dsm-firmenich Science & Research, Biotechnology, Alexander Fleminglaan 1, Delft 2613 AX, The Netherlands; 4 dsm-firmenich Science & Research, Analytical Sciences, Alexander Fleminglaan 1, Delft 2613 AX, The Netherlands; 5 Department of Bionanoscience, Kavli Institute of Nanoscience Delft, Delft University of Technology, Van der Maasweg 9, Delft 2629 HZ, The Netherlands; 6 DSM Biomedical, Urmonderbaan 22, Geleen 6160 BB, The Netherlands

**Keywords:** biofabrication, hydrogels, biomaterials, structural proteins, biopolymers, silk, elastin

## Abstract

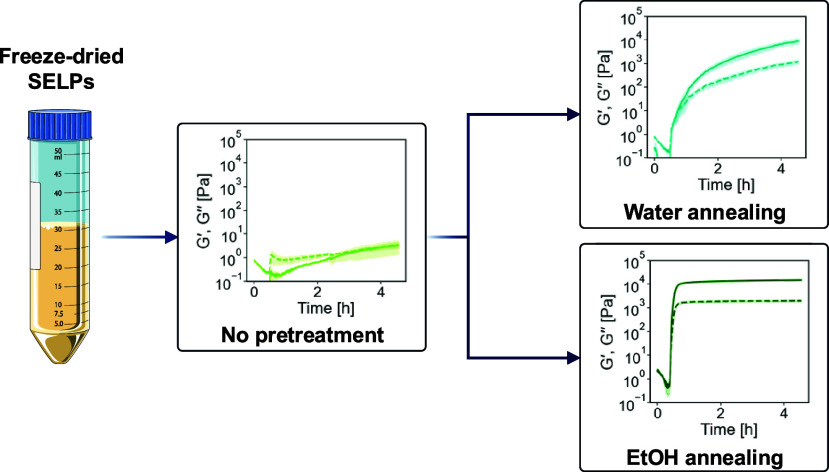

The biofabrication of recombinant structural proteins with a range of mechanical or structural features usually relies on the generation of protein libraries displaying variations in terms of amino acid composition, block structure, molecular weight, or physical/chemical cross-linking sites. This approach, while highly successful in generating a wealth of knowledge regarding the links between design features and material properties, has some inherent limitations related to its low throughput. This slows down the pace of the development of *de novo* recombinant structural proteins. Here, we propose an approach to tune the viscoelastic properties of temperature-responsive hydrogels made of silk-elastin-like polypeptides (SELPs) without modifying their sequence. To do so, we subject purified SELPs to two different postprocessing methods—water annealing or EtOH annealing—that alter the topology of highly disordered SELP networks via the formation of ordered intermolecular β-sheet physical cross-links. Combining different analytical techniques, we connect the order/disorder balance in SELPs with their gelling behavior. Furthermore, we show that introducing a functional block (in this case, a biomineralizing peptide) in the sequence of SELPs can disrupt its self-assembly and that such disruption can only be overcome by EtOH annealing. Our results suggest that postprocessing of as-purified SELPs might be a simple approach to tune the self-assembly of SELPs into biomaterials with bespoke viscoelastic properties beyond the traditional approach of developing SELP libraries via genetic engineering.

## Introduction

Nature has evolved intricate mechanisms to direct the self-assembly of structural protein biopolymers from a disordered state into ordered, complex and functional materials such as bone, collagen, silk, elastin, or mussel threads.^1^ These materials typically display impressive mechanical properties, a hierarchical structure, dynamic responsiveness, and environmental adaptability, that are conveyed by nano- and mesoscale motifs (e.g., β-sheets, helices, nanofibrils) encoded in the amino acid sequence of their constituent protein biopolymers.^2^ Additional benefits of protein biopolymers include sustainability, ease of processability, and biodegradability. Oftentimes, the self-assembly of protein biopolymers into macroscopic materials involves a phase transition from disordered protein solutions into stable phase-separated condensates or fibers.^3^ This phase transition can be tuned by controlling bottom-up interactions at the molecular scale (e.g., balance of ordered/disordered, hydrophobic/hydrophilic, or charged/neutral blocks in the protein sequence)^2^ and top-down processing conditions (e.g., ionic strength, pH, shear).^4^

Inspired by these natural fabrication strategies, scientists are increasingly using structural protein biopolymers to manufacture synthetic self-assembling technical materials with applications in medicine, food, adhesives, textiles, or membrane technology, to name a few.^5−9^ Protein biopolymers are normally harvested from animal sources (e.g., silk from silkworm cocoons, collagen from animal tissue, β-lactoglobulin from milk, lysozyme from egg whites), but these sources suffer from batch-to-batch variability, presence of contaminants, and cultural or religious concerns that limit their commercialization potential. Fortunately, developments in recombinant DNA technology and microbial bioprocesses allow us to overcome these issues and biomanufacture nonanimal-derived structural proteins. Additionally, recombinant DNA technology allows us to explore protein sequences beyond those selected for by evolution–we can now design entirely new structural proteins with properties inspired by natural structural proteins (e.g., the strength of silk, the stimuli-responsiveness of elastin, the stiffness of collagen), but that do not exist in nature.^10^ This allows us to bring together the reproducibility of synthetic polymers with the biocompatibility and sustainability of natural biopolymers.^11^

Elastin-like polypeptides (ELPs) are an archetypical example of nature-inspired recombinant structural proteins. ELPs are intrinsically disordered polypeptides (IDPs) inspired by the VPGXG tandem repeats (being X any amino acid except proline) that confer thermoresponsiveness to natural tropoelastin.^12^ ELPs have a reversible lower critical solution temperature (LCST) behavior, meaning that they are in a disordered water-soluble state below a transition temperature T_t_, but coacervate and phase separate above it.^13^ This thermoresponsiveness allows us to combine conformational freedom in the solvated state with the ability to create macromolecular hydrogel networks upon phase separation Furthermore, the thermoresponsiveness of ELPs allows us to purify them via temperature cycling, a scalable process that is cheaper than other protein purification methods like affinity chromatography.^14^

In nature, β-sheets act as physical cross-links to increase the strength of protein-based materials such as silk or amyloids.^15^ This has inspired the fusion of elastin-like blocks (VPGXG) with silk-like blocks capable of forming β-sheets (GAGAGS) using recombinant DNA technology. This approach has led to the emergence of a broad class of proteins termed silk-elastin-like polypeptides (SELPs) that combine the stimuli-responsiveness of elastin-like blocks with the enhanced structural stability and mechanical properties of silk-like blocks.^16^ SELPs hydrogels have found applications in areas like drug delivery, gene therapies, or tissue engineering.^17−23^ However, the molecular mechanisms that regulate the structure, stimuli-responsiveness and viscoelastic properties of SELP hydrogels are still unclear. Recent studies have indicated that the assembly of silk-like β-sheets in SELP solutions above LCST is a thermodynamically controlled process that happens at a slower rate than the kinetically controlled coacervation of elastin-like blocks.^24^ Coacervation above LCST brings silk-like blocks in close proximity, enabling their self-assembly into β-sheets that stabilize the transient and highly dynamic conformations of the elastin-like blocks.

Efforts to engineer the properties of SELP hydrogels have mostly focused on engineering their amino acid sequence, the molecular weight (MW) and/or the ratio of silk-like (ordered) to elastin-like (disordered) blocks.^16,24−26^ This approach has also been applied to other recombinant structural proteins, such as squid-ring teeth proteins,^27^ collagen-silk-like polypeptides,^28^ resilin-like proteins,^29^ or fusions of ELPs with partially ordered peptides.^30^ This has enabled the manufacture of hydrogels with a wide range of sizes, morphologies, and mechanical properties, albeit at the expense of lengthy and costly design-build-test-learn (DBTL) cycles. Here, we propose an alternative route to tune the mechanical and structural properties of SELP hydrogels. By subjecting purified SELPs to water annealing or ethanol (EtOH) annealing, we were able to alter the β-sheet content and thus the order/disorder balance in SELPs prior to hydrogel formation. This dramatically affected the ability of SELP solutions to form free-standing hydrogel networks above LCST. The effect of these post-treatments on the self-assembly of these proteins was assessed for two SELP variants: one containing only structural elastin- and silk-like blocks, and another containing also a functional peptide (in this case, a biomineralizing peptide). Our results bridge disorder-to-order transitions at the molecular scale, hierarchical self-assembly pathways, and macroscopic mechanical properties. In doing so, we demonstrated a previously unreported approach to engineer the viscoelasticity of SELPs hydrogels, manipulating the topology of the starting polymer network rather than the SELP amino acid sequence. This approach is simple and scalable and could accelerate the manufacture of SELP materials with a wider range of mechanical properties.

## Results and Discussion

### Sequence Design, Expression, and Purification

**Figure 1:**
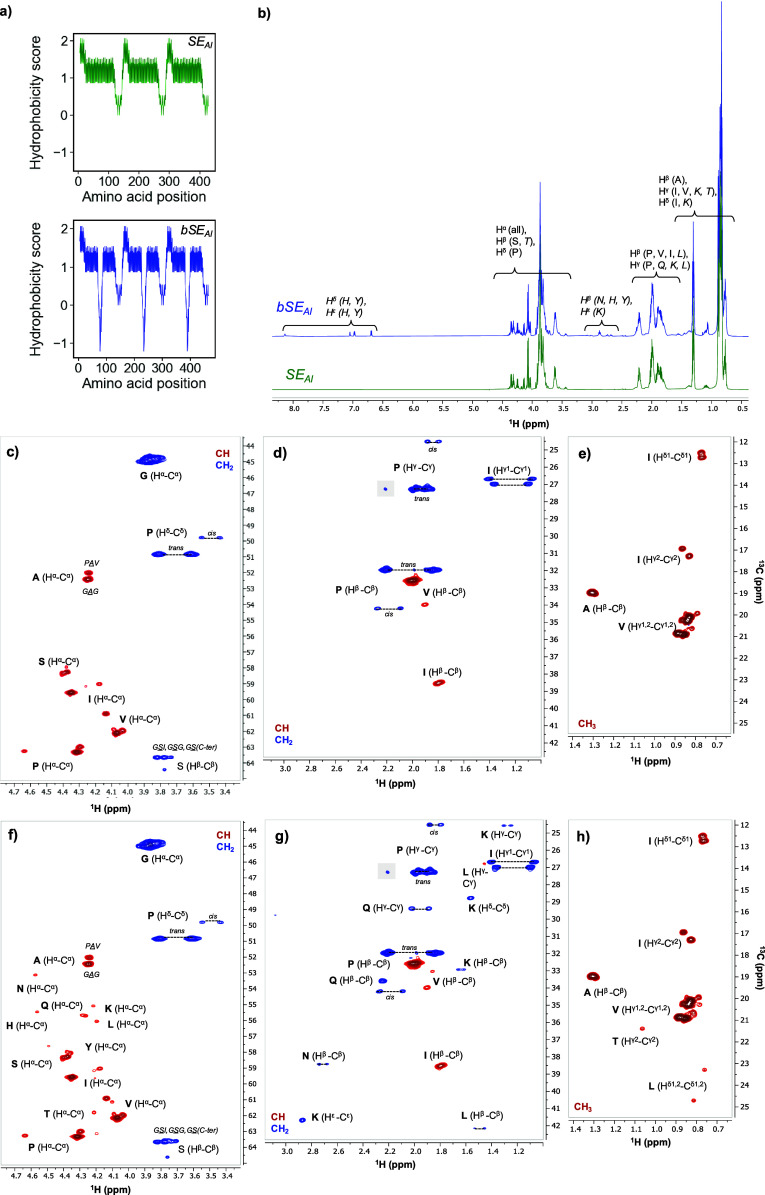
**NMR spectra of solutions of freeze-dried SE**_**AI**_**and bSE**_**AI**_. (a) Hydropathy plots of the SELP sequences tested in this work as calculated using the Kyte-Doolittle scale. (b) 1D ^1^H NMR spectra of SE_AI_ (green, bottom) and bSE_AI_ (blue, top) show highly pure proteins. Spectral regions are labeled with the specific protons that give signals in these regions, using labels α–ε to indicate the position in the amino acid and the amino acid one letter codes in normal font for the residues in elastin/silk-like blocks (V, I, S, G, A, P) and italic for the residues specific for the biomineralizing domain (H, Y, N, K, L, T, Q). (c-h) Zooms of the 2D edited ^1^H–^13^C HSQC spectra of SE_AI_ (c-e) and bSE_AI_ (f-h) show specific signals for residues in different blocks in the protein. Signals of CH and CH_3_ groups are positive (red) and signals from CH_2_ groups negative (blue). Spectral artifacts due to baseline distortions are indicated in gray. Thresholds of the different zooms have been adapted for clarity. Full spectra are shown in the Supporting Information.

The sequences of SELPs used in this study are shown in Table 1. SE_AI_ and bSE_AI_ were designed with a segmented copolymer structure that combined flexible/crystalline, structural/functional, and hydrophobic/hydrophilic blocks (Figure 1a). Both SELPs contained the same type and number of structural blocks, which were inspired by the sequences of tropoelastin (VPGVG, VPGIG, and IPAVG) and silk fibroin (GAGAGS). The high content in proline and glycine of elastin-like blocks promotes hydration and a disorder structure for SELPs, resulting in high conformational freedom and rubber-like elasticity.^16,31^ VPGVG and VPGIG are inspired by the canonical ELP building block VPGXG^12^ and display fully reversible thermoresponsiveness, whereas IPAVG leads to faster and kinetically arrested gelation^25,32^ and displays hysteresis between solvation and desolvation. In turn, GAGAGS silk-like blocks can form ordered β-sheets that behave as intra- or intermolecular quasi-irreversible physical cross-links, enhancing the structural stability and mechanical properties of hydrogels. Additionally, we studied the effect of a functional (nonstructural) block on the self-assembly of SELPs. To do so, we introduced a hydrophilic block that has been reported to nucleate the biomineralization of hydroxyapatite (VTKHLNQISQSY)^33^ into the structure of bSE_AI_.

**Table 1:** Name, Amino Acid Sequence, and Theoretical MW of the SELP Library

**name**	**sequence**	**theoretical MW (kDa)**	**experimental MW (kDa)**
SE_AI_	[(IPAVG)_4_[(VPGVG)_2_(VPGIG)(VPGVG)_2_]_4_(GAGAGS)_4_]_3_	34.9	34.8
bSE_AI_	[(IPAVG)_4_[(VPGVG)_2_(VPGIG)(VPGVG)_2_]_2_VTKHLNQISQSY[(VPGVG)_2_(VPGIG)(VPGVG)_2_]_2_(GAGAGS)_4_]_3_	39.1	39.2

SE_AI_ and bSE_AI_ were recombinantly expressed in *E. coli* and purified via inverse transition cycling.^34^ The purity of as-purified SELPs was confirmed via proton 1D solution nuclear magnetic resonance (NMR) (Figure 1b) by analyzing SELP solutions in D_2_O at 295 K. The spectra confirmed the high purity of both SELPs, with sharp signals indicating a highly mobile protein backbone. The signals for both SELPs had a similar profile, with the exception of the signals for the biomineralizing block, which were only found in bSE_AI,_. Additional details were gathered via natural abundance 2D ^1^H–^13^C spectra (Figure 1**c-h** and Figures S1–S2). Signals were resolved through their adjacent carbon atom, resulting in a cross-peak at a specific ^1^H and ^13^C frequency. Interestingly, the 2D spectra of the two SELPs again overlapped perfectly for amino acids in the common building blocks for both proteins. This indicated that the biomineralizing block did not alter the structure of the elastin-like and silk-like blocks. Putative assignment of the different cross-peaks was done using the chemical shifts for the different amino acids in a protein as provided by the Biological Magnetic Resonance Data Bank (BMRB)^35^ and by Wang & Jardetzky.^36^ Exact assignment of the cross-peaks for the H^α^-C^α^ region was challenging and labels were placed where the signals were expected. For other regions, cross-peaks could easily be attributed to a specific C–H couple in one of the amino acids. Purified SELPs were highly disordered in solution, as shown by the presence of chemical shifts typical of both cis and trans conformations (Figure 1d and 1**h**) in proline residues–a feature typical in elongated unstructured proteins.^37,38^ Other residues also displayed multiple signals for their backbone and side-chain CH couples. For instance, two populations of alanine H^α^-C^α^ signals were observed, which were putatively assigned to alanine in either the PAV or GAG sequence (see Figure 1c and 1**g** for SE_AI_ and bSE_AI_, respectively). Similarly, valine residues also showed multiple signals, where especially the main cross-peak (2.0 and 32.5 ppm) for the H^β^-C^β^ correlation was compatible with valine in a random coil structure, while the minor signal at 1.9 and 34 ppm was compatible with valine in a sheet-like structure (Figure 1d and 1**h**).^36^

**Figure 2:**
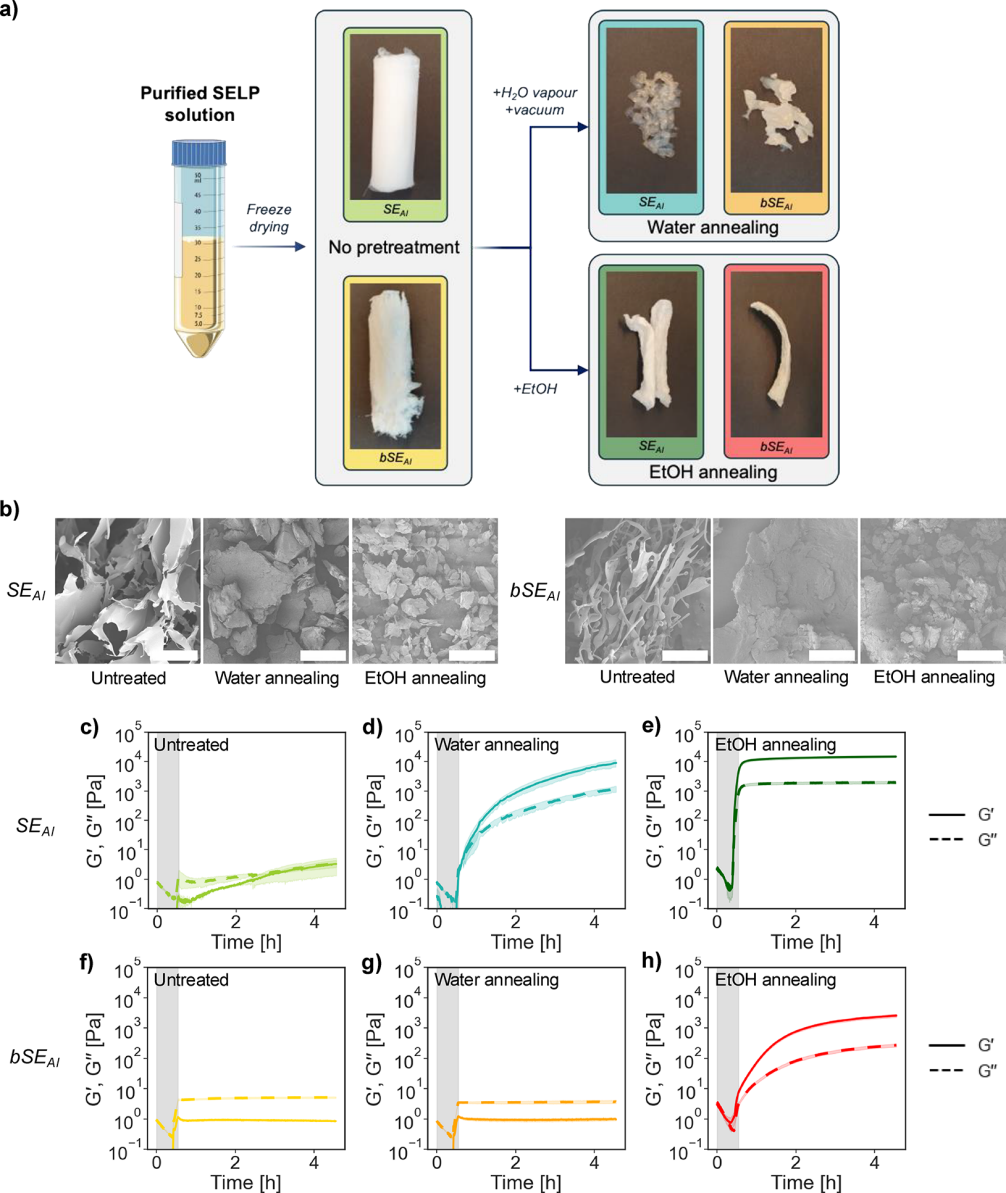
**SELP formulations, macro- and microscopic appearance, and rheological properties.** (a) Scheme of the different formulations tested in this work, with pictures of the resulting, dry material for each formulation. (b) SEM pictures of the microstructures for each SELP formulation (scale bar = 200 μm). (c-h) Rheological characterization (storage modulus *G*′ and loss modulus *G*′′) of SELP solutions in Milli-Q water (15 wt %) for untreated, water annealed and EtOH annealed samples during a temperature sweep between 4 and 37 °C (gray background) at a rate of 1 °C/min, followed by a time sweep at 37 °C for 4 h (white background) (*n* = 2).

The properties of SE_AI_ and bSE_AI_ were tested in three different formulations: (1) untreated, (2) after postprocessing via water annealing and (3) after postprocessing via EtOH annealing. The water and EtOH annealing methods have been widely used to tune the amount of intermolecular β-sheet physical cross-links formed in silk-based materials after material synthesis.^39,40^ Here we applied them to dry, purified SELPs prior to material synthesis. Our hypothesis was that by altering the content of β-sheets in these materials, we could tune their ability to form hydrogels in a thermoresponsive manner, as well as their viscoelastic properties. The three formulations tested in this work are displayed in Figure 2a, showing the clear changes in the physical appearance of SELPs caused by both types of annealing. Untreated SELPs were obtained directly after freeze-drying and displayed a porous structure with a spongy consistency. Conversely, water annealed and EtOH annealed samples became rigid and with a plastic-like consistency. Annealing postprocessing heavily modified the microstructure of SELPs, as observed via SEM (Figure 2b), but did not alter their MW, as shown by SDS PAGE (Figure S3). Annealing also affected the solubility of SELPs in water: while untreated SELPs were fully soluble at concentrations in the range of 0.5–250 mg/mL, water and EtOH annealed SELPs required cryomilling to render them soluble in Milli-Q water.

### Rheology

The rheological behavior of SE_AI_ and bSE_AI_ solutions (15 wt %) was probed via small amplitude oscillatory rheology (Figure 2**c-h**). The linear viscoelastic (LVE) region of these solutions was determined via amplitude sweeps after 4 h of incubation at 37 °C (Figure S4). Most of the formulations showed a response independent of strain amplitude (0.01–15%). Only solutions from water annealed and EtOH annealed SE_AI_ showed a decrease at high amplitudes (above 1% for water annealed SE_AI_, and above 8% for EtOH annealed SE_AI_). SELP solutions were also subjected to a temperature ramp (1 °C/min) between 4 and 37 °C (gray background areas in Figures 2c–h), followed by a time sweep at 37 °C for 4 h (white background areas in Figures 2c–h). Untreated SE_AI_ showed a slow but steady increase in the storage modulus once the T_t_ was surpassed (Figure 2c). This was attributed to the continuous formation of intermolecular β-sheets for SELP solutions above LCST. To confirm this, SE_AI_ solutions in 6 M GdmCl were assessed, due to the ability of GdmCl to disrupt β-sheets in protein networks.^41^ Indeed, the addition 6 M GdmCl eliminated the increase in storage modulus over time above LCST (Figure S5), confirming that β-sheets (and not just protein entanglements) were the cause of the increase in modulus of untreated SE_AI_ solutions above LCST. Water-annealed SE_AI_ solutions showed a steady increase in *G*′ during the temperature and time sweeps akin to that of untreated SE_AI_, but at a much faster rate (Figure 2d). Finally, EtOH-annealed SE_AI_ solutions gelled instantly once T_t_ was surpassed, and its storage and loss moduli remained unchanged thereafter (Figure 2e). These data confirms that postprocessing indeed altered the ability of SE_AI_ solutions to self-assemble into percolated hydrogel networks above T_t_.

The self-assembly of bSE_AI_ was negatively impacted by the presence of the hydrophilic biomineralizing functional block. Untreated and water-annealed bSE_AI_ were unable to gel after 4 h at 37 °C. (Figure 2**f,g**) Furthermore, unlike SE_AI_, the storage modulus (*G*′) of bSE_AI_ solutions only increased until T_t_ was surpassed, remaining constant and lower than the loss modulus (*G*′′) thereafter. Only EtOH annealing rendered bSE_AI_ able to gel, with a gelling profile similar to that of water annealed SE_AI_ (Figure 2h). The different formulations tested here also led to wide variations in the stress relaxation spectra of SELP solutions (Figure S6). The solutions that were unable to gel exhibited a dependence of the shear moduli with frequency, likely due to the ability of those networks to relax entanglements because of their low content in intermolecular β-sheets.

**Figure 3:**
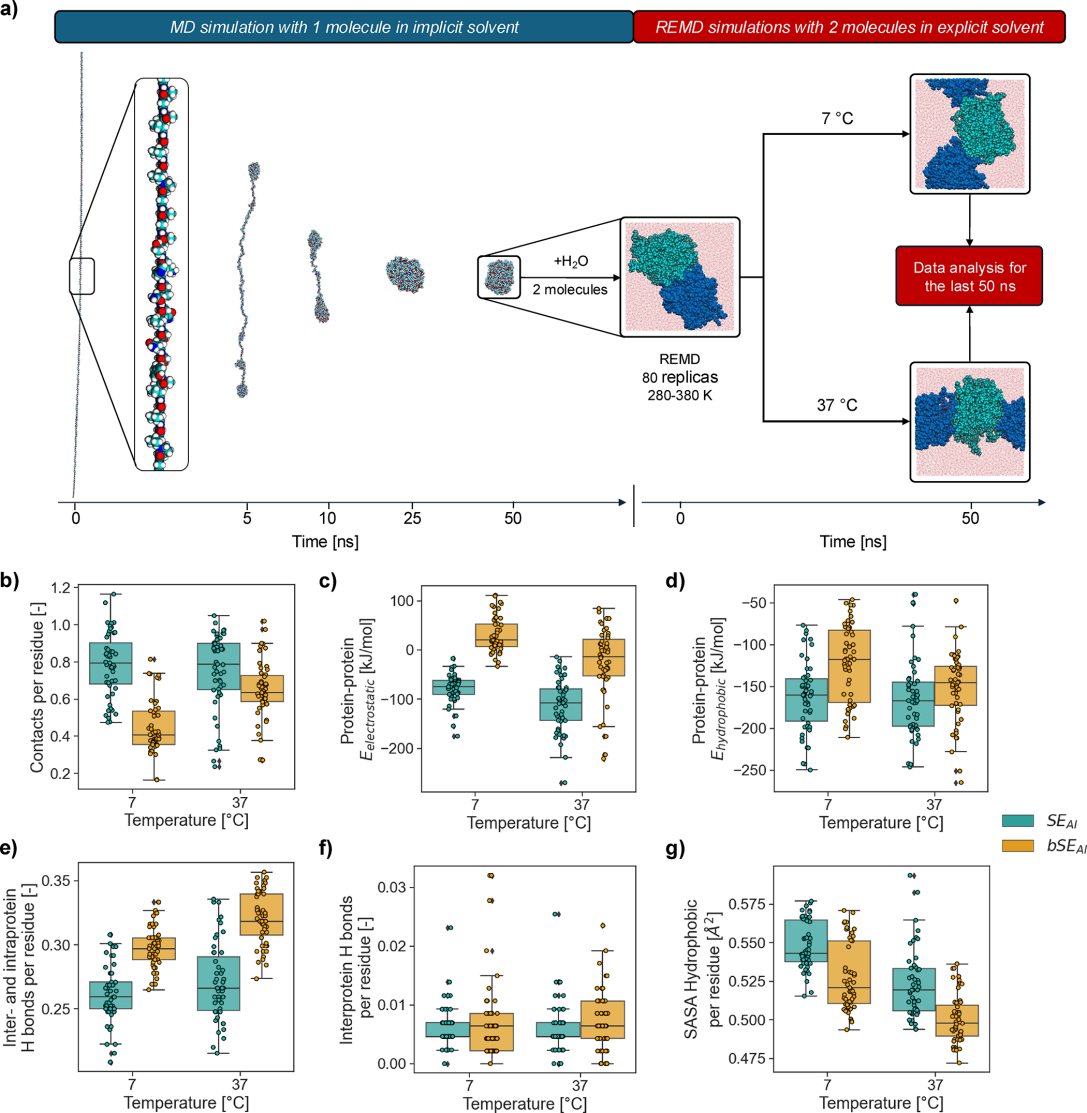
**REMD simulations of SELPs.** (a) Screenshots of the folding process (one chain in implicit solvent) and REMD simulations (two chains in explicit solvent) for bSE_AI_. (b) Interprotein contacts normalized by the number of residues in each SELP. (c) Electrostatic interprotein interaction energy. (d) Hydrophobic interprotein interaction energy. (e) Total protein hydrogen bonds normalized by the number of residues in each SELP. (f) Interprotein hydrogen bonds normalized by the number of residues in each SELP. (g) Solvent accessible surface area of hydrophobic residues normalized by the number of residues in each SELP.

To investigate the differences in gelation between SE_AI_ and bSE_AI_, replica exchange molecular dynamics (REMD) simulations were performed. These simulations aimed to capture the differences in interchain interactions of elastin-like blocks in both SELPs below T_t_ (7 °C) and above T_t_ (37 °C). Effects arising from the water or EtOH annealing post-treatments were not considered in these simulations, as those post-treatments aimed at controlling the formation of β-sheets via silk blocks but are not expected to affect the thermoresponsiveness of elastin-like blocks. REMD simulations were performed with systems consisting of two molecules of SE_AI_ or bSE_AI_ (Figure 3a). Overall, the simulations indicated a lower propensity of bSE_AI_ to interact with neighboring chains. SE_AI_ molecules had a higher propensity to form contacts with each other than bSE_AI_, especially at 7 °C (Figure 3b). These contacts (defined as α carbons in the backbone of different SELP chains located less than 10 Å apart) are a necessary step for the formation of interprotein β-sheets, which are critical for the formation of robust hydrogel networks in SELPs. The higher stability of SE_AI_-SE_AI_ interactions was also shown by their more negative values of the electrostatic interaction energies (and to a lesser extent hydrophobic interaction energies too) (Figures 3**c-d**). Additionally, bSE_AI_-bSE_AI_ systems formed more hydrogen bonds (normalized by the number of residues of each SELP) (Figure 3e). However, the number of interprotein hydrogen bonds was similar for both systems (Figure 3f), indicating that the increase in hydrogen bonding in bSE_AI_-bSE_AI_ systems was due to intraprotein hydrogen bonds. Additionally, the solvent accessible surface area (SASA) of hydrophobic amino acids was lower for bSE_AI_ (Figure 3g). A lower SASA for hydrophobic amino acids is detrimental for the formation of robust SELP hydrogels, as contacts between hydrophobic amino acids are key for the coacervation of elastin-like blocks.^31,42^

### Network Structure

The SELPs solutions described in Figure 2c were all prepared in the same manner: dry SELPs were dissolved in Milli-Q water (15 wt %), incubated on ice for 10 min with occasional stirring, and subsequently analyzed by small amplitude oscillatory rheology. This suggested that the variations in the behavior of each formulation must arise from features already present in the dry SELPs. To test this idea, we applied a range of analytical techniques, including DLS, FTIR, AFM, SAXS, and WAXS.

**Figure 4:**
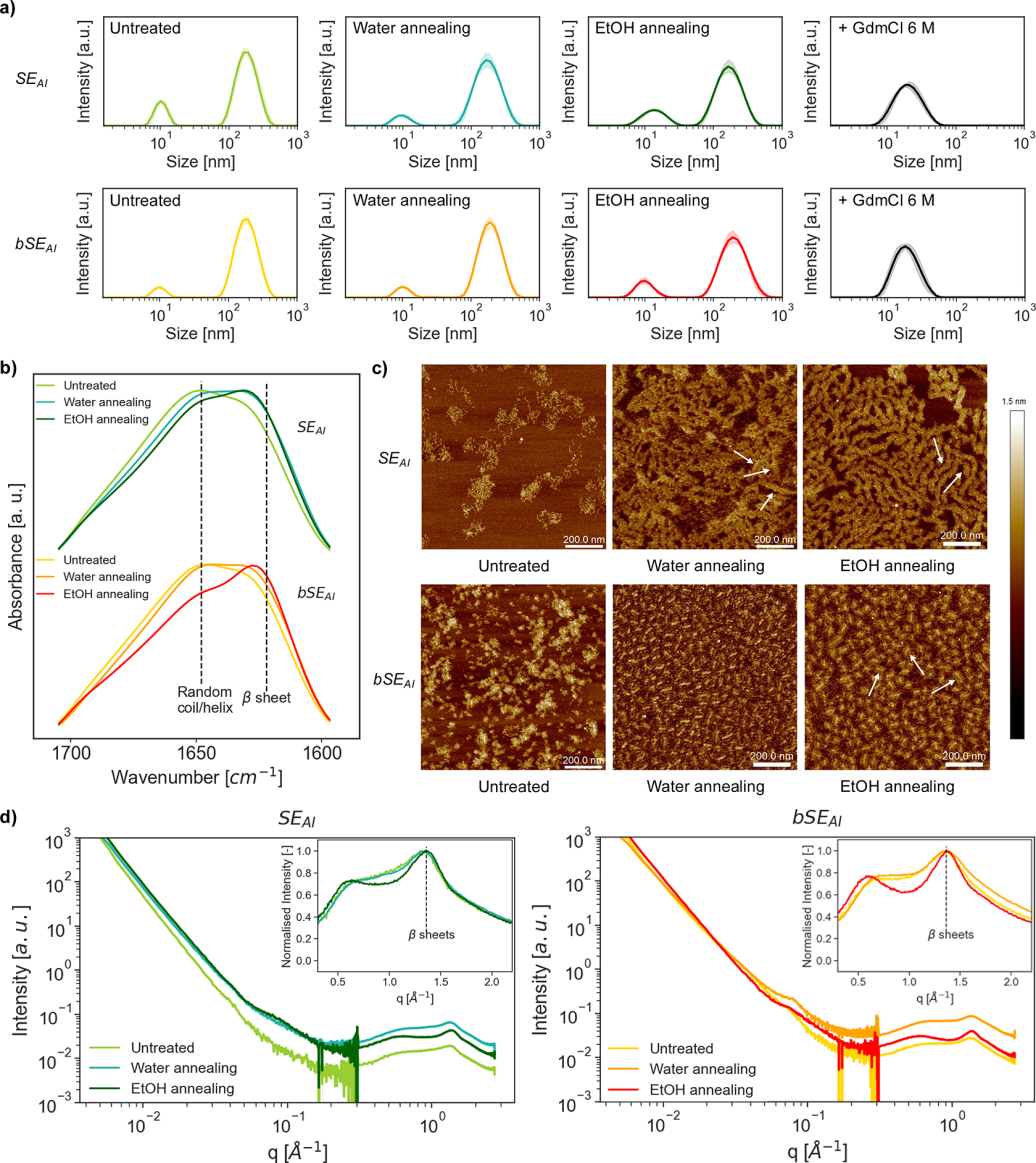
**Characterization of the network structure of SELPs in different formulations.** (a) Size distributions of the hydrodynamic radius (*n* = 5) of 0.5 mg/mL solutions in Milli-Q water of the different SELP formulations tested in this work. (b) Normalized FTIR spectra of the amide I region for dry SELP formulations (*n* = 2). (c) AFM images of SELP networks in different formulations, imaged in dried conditions on mica. (d) SAXS/WAXS patterns for SELPs. Insets represent the normalized diffraction pattern for WAXS measurements to facilitate the comparison in terms of β-sheet content for each formulation.

The hydrodynamic diameter *D*_h_ of SE_AI_ or bSE_AI_ was assessed via DLS using diluted solutions (0.5 mg/mL) (Figure 4a). Before analysis, the samples were passed through a 0.2 μm syringe filter to remove any dust particles, followed by an equilibration at 25 °C (below T_t_) for 5 min. The solutions showed a peak in the range of the expected values for the *D*_h_ of single IDP molecules in solution (10.9 nm for SE_AI_ and 11.4 nm for bSE_AI_). Surprisingly, all samples contained a second, more intense peak at higher *D*_h_ values, an effect previously observed for other SELPs.^43^ Unfortunately, the reason for this (given that the samples passed through a 0.2 μm filter before analysis) remains unclear at this stage. We hypothesize the larger *D*_h_ values might originate from intermolecular β-sheets that could form below T_t_ due to the shearing flow in the syringe filter, or from random contacts between silk-like blocks in solution. One argument that supports this hypothesis is that SELP solutions in GdmCl 6 M do not exhibit such large particles. We consider it unlikely that these aggregates arise from the thermoresponsive aggregation of SELPs, because these experiments were carried out below T_t_.

We next tested the effect of the SELP formulation (untreated, water-annealed, or EtOH-annealed) on its secondary structure via Fourier Transform Infrared (FTIR) spectroscopy (Figure 4b, full spectra in Figure S7). The annealing postprocessing caused a shift of the amide I band toward the region around ca. 1620 cm^–1^, a fingerprint of the formation β-sheets in protein materials.^44^ The prominence of the β-sheets band followed the order untreated < water annealing < EtOH annealing for both SELPs. Nonetheless, despite the increasing content in β-sheets, the polymers retained a significant fraction of disordered regions, as shown by peak/shoulder at around 1650 cm^–1^. The effect of postprocessing was also clearly visible via AFM imaging (Figure 4c and Figure S8). Both SE_AI_ and bSE_AI_ without postprocessing contained isolated clusters of disordered protein chains. In turn, water and EtOH annealing caused the emergence of fibrillar structures formed by stacked layers perpendicular to the direction of the fiber (as exemplified by the arrows displayed in Figure 4c). The change in direction of those arrows evidenced that the observed layered structures did not arise from artifacts due to the scanning direction of the tip. Notably, the growth of those stacked structures was affected by the type of SELPs: while SE_AI_ was able to form long fibrillar structures, bSE_AI_ did not grow beyond small wormlike micelles. The type of annealing affected the morphology of those fibers: EtOH annealing formed longer fibers in SE_AI_, and wormlike micelles of a higher radius in bSE_AI_. Furthermore, water-annealed SE_AI_ samples also showed the presence of a significant amount of small fibrillar structures (Figure S9) that were not present in EtOH-annealed samples. This hinted that EtOH annealing was more effective in forming β-sheets (as indicated also by FTIR), resulting in a network with higher connectivity and a more ordered structure.

X-ray scattering was used to gain further insight into the nanoscale structures present in dry SELPs. As expected, WAXS diffraction patterns confirmed the presence of ordered β-sheet regions in all the samples analyzed (Figure 4d). The presence of diffuse rings in the 2D WAXS patterns was consistent with the polymers being mainly disordered and without a preferential orientation for the β-sheet regions (Figure S10). Radial integration of the 2D scattering profiles showed a peak at q = 1.30–1.38 Å^–1^ for all formulations in both SELPs. According to the Bragg expression,^45^ this corresponded to d = 2π/q = 4.55–4.85 Å, which was within the typical range of distances reported for adjacent β-strands in crystalline regions for protein materials.^46−48^ Similar patterns were reported for other protein-based materials, such as silk-amyloid fusions,^49^ whey-PHPA elastomers,^50^ tropoelastin-based materials^51^ or plant-based thermosetting materials.^46^ The increased sharpness of the peak at q = 1.30–1.38 Å^–1^ for water- and EtOH-annealed samples again confirmed the higher content of β-sheets in these materials. The annealing postprocessing did alter the content in β-sheet crystals, but only caused small variations in their size. The monotonic decrease with increasing wavenumber q of the SAXS intensity curves of SE_AI_ and bSE_AI_ and their lack of marked diffraction peaks (Figure 4d and Figure S11) again confirms that both materials were also mainly disordered and had no characteristic length scale, regardless of the formulation under consideration. Only a small shoulder at q ∼ 0.07 Å^–1^ was observed in annealed samples, which could be attributed to the formation of nanocrystalline domains.

Taken together, the results from different analytical techniques provide a picture of how postprocessing routes can be leveraged to tune nanoscale features (i.e., intermolecular β-sheets) in SELPs, and how those features influence the macroscopic gelation of SELP solutions. Untreated SELPs in solution were highly flexible and had limited connectivity. As a result, they phase-separated above T_t_ but could not form a cohesive network. Water- or EtOH-annealed SELPs had an increased content in intermolecular β-sheets. These β-sheets appeared to catalyze the self-assembly of additional β-sheet, especially above T_t_ thanks to the coacervation of elastin-like blocks. This would explain the increase in storage modulus observed for water-annealed SE_AI_ or EtOH-annealed SE_AI_ and bSE_AI_ (compared to untreated formulations). The rate at which new β-sheets formed above T_t_ appeared to be influenced controlled by the initial content of β-sheets in dry SELPs. As more β-sheets formed, they stabilized the nascent hydrogel network via strong hydrogen bonding, until a saturation level was achieved. This would be consistent with nucleation-aggregation logistic mechanisms akin to those proposed for the self-assembly for natural and synthetic silk materials:^48,52,53^ the higher the β-sheet content in the starting material, the faster the saturation level is achieved.

Overall, the data suggests the existence of a percolation threshold in the connectivity of SELP chains via β-sheets above which SELP solutions at *T* > T_t_ can form a robust hydrogel. Given that SELPs are typically purified using alternating low-temperature and high-temperature cycles, our results underscore how β-sheets formed between silk-blocks during the high-temperature cycles can dramatically impact the gelling behavior and the viscoelastic properties of SELP materials. Moreover, the properties of SELPs have been traditionally tuned by modifying the sequence, MW or block composition.^16,24,43^ However, such an approach requires long DBTL cycles of gene synthesis, transformation, protein expression and purification, before the effect of those modifications can be evaluated. Our results hint at a simpler and controllable strategy to tune the gelling propensity of SELPs–by merely adjusting the amount of β-sheets in the starting material via water or EtOH annealing–that could accelerate DBTL cycles for new protein-based materials.

### Reversibility

**Figure 5:**
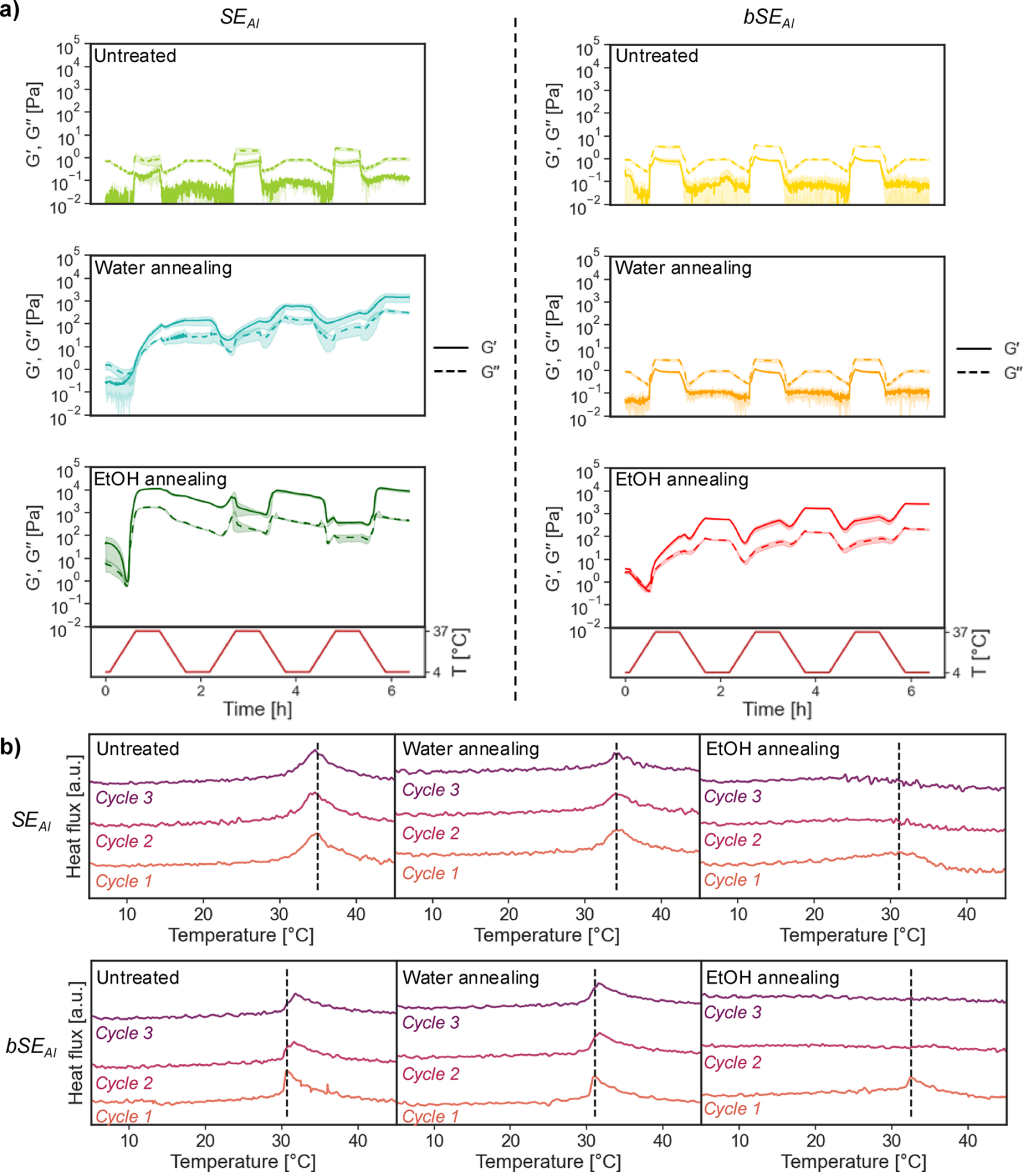
**Influence of the formulation on the reversible gelation of SELPs.** (a) Thermal cycling between 4 and 37 °C of SELP solutions (15 wt %) at a heating/cooling rate of 1 °C/min (*f* = 1 Hz, γ = 0.3%), with 30 min of resting time between temperature ramps (*n* = 2, SE_AI_ on the left column, bSE_AI_ on the right column). (b) Thermograms for 3 thermal cycles between 4 and 50 °C (1 °C/min) of SELP solutions (15 wt %). *Y*-axis is offset for clarity. The vertical dashed line indicates the location of the thermogram maximum (associated with the T_t_) for cycle 1.

We assessed the reversibility of the gelation of SELP solutions over three temperature cycles between 4 and 37 °C, with a resting time of 30 min after each temperature ramp (Figure 5a). In previous studies, we described the fully reversible gelation for ELP solutions with the same concentration.^25^ A similar behavior was described elsewhere when cycling SELP solutions too, but only for a few temperature cycles.^43^ Once the number of cycles increased (>10 cycles), the thermoresponsiveness of SELP hydrogels disappeared. In our work, the reversibility of the gelation for the SELP solutions was strongly dependent on the initial amount of β-sheets and the presence of a biomineralizing block. Untreated SE_AI_ and bSE_AI_ were unable to gel after three temperature cycles, with *G*′′> *G*′ throughout. Water-annealed SE_AI_ showed an onset for the gelation once T_t_ was surpassed during the first ramp. From that point onward, a network formed and *G*′ dominated over *G*′′ regardless of the temperature, even during cooling ramps, and the moduli increased with every heating ramp from 4 to 37 °C. In turn, water-annealed bSE_AI_ was unable to gel after three temperature cycles, demonstrating the deleterious effect of the biomineralizing block for self-assembly. EtOH-annealed samples gelled during the first heating ramp for both SE_AI_ and bSE_AI_. However, their behavior in subsequent cycles was different. SE_AI_ achieved its maximum *G*′ as soon as T_t_ was surpassed in the first heating ramp, whereas bSE_AI_ kept increasing its moduli during the three cycles.

Notably, a loss of the thermoresponsiveness was observed for EtOH-annealed SE_AI_ solutions after the first temperature cycle. In fact, EtOH annealed SE_AI_ networks became softer above T_t_ during the second temperature ramp between 4 and 37 °C. This was different from the typical behavior of solutions with LCST-like behavior, which are normally softer below T_t_ than above it. A possible explanation for this behavior is provided by the theory of energy landscape:^54,55^ flexible elastin-like blocks are able to explore many local energy minima due to their conformational freedom, whereas silk-like blocks evolve toward global energy minima represented by β-sheets. Once the amount of intermolecular β-sheets reaches a critical threshold, they impose a high energy barrier on the conformational freedom of elastin-like block. Thus, we hypothesize that EtOH annealed SE_AI_ networks fully developed during the first temperature cycle, reaching a saturation value for the β-sheets content that restricted the conformational freedom of elastin-like blocks, effectively erasing their thermoresponsive behavior. The resulting behavior was more akin to that of rubbers, in which higher temperatures cause a relaxation of entanglements that weakens their mechanical properties.^50^ We also observed that the biomineralizing blocks in bSE_AI_ solutions hindered their self-assembly into robust hydrogels, delaying the formation of a fully developed network. We hypothesize that this could be prevented in future DBTL cycles by changing the location of the biomineralizing blocks, for instance by placing them at the ends of the SELPs–rather than regularly distributing them throughout the sequence.

The reversibility of gelation was also analyzed via DSC, cycling 15 wt % solutions of the various SE_AI_ and bSE_AI_ formulations between 4 and 50 °C for three times (Figure 5b). The thermograms for untreated SE_AI_ and bSE_AI_ showed a similar behavior, with a peak at around 35 °C arising from the T_t_ and consequent coacervation of elastin-like blocks. In water-annealed samples, the first cycle had a more prominent peak than the subsequent cycles, especially for SE_AI_. This indicated that an initial hydrogel network formed during the first cycle, and that every subsequent cycle progressively decreased the thermoresponsiveness of elastin-blocks (as shown by the decreasing the intensity of the peak associated with T_t_). For EtOH-annealed samples, the T_t_ peak was only present in the first cycle. The thermogram of subsequent cycles displayed a flat profile, indicating that the thermoresponsiveness of elastin-like blocks disappeared. Overall, the rheology and DSC results underscored the critical role of silk-like blocks not only in controlling the robustness of SELP hydrogel networks, but also their thermoresponsiveness.

## Conclusions

The genetic basis of sequence and length control in SELPs has resulted in a wealth of studies that investigate the effect of amino acid composition, MW, block composition or physical/chemical cross-linking strategies on the gelation of SELP solutions. Here we proposed an alternative route to tune the viscoelasticity and gelling propensity of SELP solutions by applying a postprocessing step (water annealing or EtOH annealing). These postprocessing steps significantly altered dry SELPs after purification, influencing their connectivity via intermolecular β-sheets, which also impacted the gelling propensity. Using molecular, micro-, and mesoscale experimental and computational characterization techniques, we provided a comprehensive view of the complex crosstalk between ordered silk-like blocks and flexible and stimuli-responsive elastin-blocks. Water and EtOH annealing increased the β-sheet content in SELPs, with the higher content of β-sheets in EtOH-annealed samples enhancing their ability to gel. Our results also underscored the deleterious effect of a functional building block (in this case, a biomineralization block) on the gelation of SELPs. The postprocessing methods used here did not require the use of toxic solvents, covalent cross-linkers, or nonbiodegradable materials. Moreover, they provided a facile top-down approach to direct the bottom-up self-assembly of SELPs. Overall, this work suggests a new route to tune the supramolecular self-assembly of SELPs by tuning the order–disorder balance after protein purification, rather than by sequence engineering. While this approach is demonstrated here for SELPs, it could be applicable to other protein-based materials that rely on β-sheets to control their mechanical properties, such as materials derived from plant proteins^56^ or squid ring teeth proteins.^57^

## Methods

### Synthesis and Microbial Production of the SELP Library

Two SELP sequences (SE_AI_ and bSE_AI_) were designed (Table 1). SE_AI_ was formed entirely by structural silk-like blocks (GAGAGS) or elastin-like blocks (IPAVG, VPGVG, and VPGIG), whereas bSE_AI_ also contained a functional domain capable of biomineralizing hydroxyapatite (VTKHLNQISQSY). Synthetic DNA sequences encoding for the full length of the SELPs were purchased from GeneArt (Regensburg, Germany). Plasmids containing the SELP genes were then transformed into an electrocompetent *E. coli* K12 strain proprietary of DSM (Delft, The Netherlands). Successful transformants were randomly selected and used for bacterial fermentation in 2-L shake flasks containing 500 mL of Terrific Broth medium. Cultivation was performed at 37 °C and cells were induced with l-arabinose when the optical density of the culture at 600 nm reached 0.6. Cells were harvested 4 h after induction by centrifugation at 5500 rcf for 20 min at 4 °C. The supernatant was decanted, and the cell pellets were subjected to a freeze–thaw cycle to rupture them. Thereafter, cell pellets were resuspended in Milli-Q water, and the pH adjusted to 4 to prevent the cleavage by proteases. This was especially relevant for bSE_AI_, because bioactive domains can sometimes exhibit degradation by proteases.^14^ The resulting suspension was then tip sonicated on ice to release the SELPs. The LCST behavior of SELPs allowed for their purification via a simple nonchromatographic method called inverse temperature cycling, alternating between the one- and two-phase regime of their phase diagram.^14,58^ The phase-separation of SELPs was triggered by adding 2 M NaCl and incubating the solutions at 42 °C. The duration of the incubation step at 42 °C was kept to a minimum (until turbidity was observed in the solution) to reduce the formation of irreversible β-sheets between silk blocks, as these could turn the SELPs insoluble.

Purified SELPs were desalted in 3000 MWCO Amicon ultra-15 centrifugal filter units (MilliporeSigma). The desalted materials were then resuspended in Milli-Q water, flash-frozen and lyophilized, followed by storage at −20 °C until further use. The theoretical hydrophobicity of the SELPs was calculated using the Kyte-Doolittle scale.^56^

### Protein Characterization

#### Molecular Weight (MW) Determination

The average molecular weight of SELPs was determined via reversed phase LC-HRMS^59^ (Figure S12).

#### Sodium Dodecyl Sulfate–Polyacrylamide Gel Electrophoresis (SDSPAGE)

SDS PAGE was performed using NuPAGE 4–12% Bis-tris gels (Invitrogen). Mark12 unstained standard (Thermo Fisher) was used as protein ladder. Gels were stained using SYPRO Red gel staining agent (Invitrogen) following the manufacturer’s protocol.

#### NMR Spectroscopy

Lyophilized SELPs were dissolved in deuterated water (D_2_O) to a concentration of 50 mg/mL. NMR spectra of the natural abundance nuclei were recorded at 295 K on a 700 MHz spectrometer equipped with cryogenic probe. For the 1D spectrum excitation sculpting was used to suppress residual water. For the 2D ^1^H–^13^C correlation spectra a multiplicity edited sensitivity enhanced HSQC was applied using Echo/Antiecho-TPPI for phase selection.

### Protein Formulations

Purified SE_AI_ and bSE_AI_ were used in three different formulations (untreated, postprocessing via water vapor annealing, and postprocessing via EtOH annealing). These formulations led to different contents in intermolecular β-sheet physical cross-links via the silk-like blocks.

#### Untreated

SELPs were used as obtained after purification and freeze-drying.

#### Water Vapor Annealing

Freeze-dried SELPs were subjected to water vapor annealing by placing them in a vacuum chamber along with a shallow Petri dish containing 250 mL of distilled water. The SELPs did not directly contact the water. The vacuum chamber pressure was lowered to 13 mbar to create a humid environment inside the chamber at room temperature and kept overnight under those conditions. Afterward, the water-annealed SELPs were air-dried in a fume hood, cryo-milled, and stored in a closed container at −20 °C until further use.

#### Ethanol Annealing

Freeze-dried SELPs were submerged in absolute ethanol at room temperature and placed in a rolling bench overnight. Afterward, EtOH was discarded and the EtOH-annealed material was air-dried in a fume hood, cryo-milled, and stored in a closed container at −20 °C until further use.

### Hydrogel Formation and Characterization

#### Rheology

Rheological characterization was performed to assess the ability of SELP solutions to form hydrogels, and to characterize their viscoelastic properties. Dry SELPs in each formulation (untreated, after water vapor annealing, or after EtOH annealing) were dissolved in cold Milli-Q water at a concentration of 15 wt %. Small amplitude oscillatory shear rheology measurements were performed on a stress-controlled rheometer (Anton Paar MCR 301) using a cone–plate geometry with a diameter of 50 mm and a cone angle of 1°. The temperature was controlled by a Peltier system. For each test, 590 μL of freshly prepared SELP solutions were loaded at 4 °C onto the bottom plate using a pipet, followed by equilibration for 5 min. Low viscosity mineral oil (Sigma-Aldrich) was applied to the air-sample interface around the measuring geometry to prevent water evaporation. The LCST-like behavior of SELP solutions was analyzed with a temperature sweep between 4 and 37 °C (with a rate of 1 °C/min). The reversibility of gelation was assessed with three temperature cycles between 4 and 37 °C (with a rate of 1 °C/min). Holding times of 30 min were applied at 4 and 37 °C, after each temperature ramp. Strain sweeps were performed from 0.01% to 15% at a frequency of 1 Hz to evaluate the linear viscoelastic region of these hydrogels. Frequency sweeps were carried out at 4 and 37 °C from 0.1 to 100 rad/s, using a constant strain of 0.3%. Additional rheological measurements were done to separate the influence on the mechanical properties of β-sheets from those owed purely to biopolymer entanglements. To that end, 15 wt % solutions of SELPs in 6 M solutions of guanidinium chloride (GdmCl) in Milli-Q water were used.

#### Fourier Transform Infrared Spectroscopy (FTIR)

Dry SELPs were analyzed by FTIR to assess their secondary structure in the dry state for the different formulations. Infrared spectra were measured in a Bruker Vertex 70 Attenuated Total Reflectance FTIR device equipped with a Harrick split pea accessory. For each measurement, 64 scans with a resolution of 2 cm^–1^ were recorded in the range of 650 to 4000 cm^–1^.

#### Dynamic Light Scattering (DLS)

The hydrodynamic diameter of SELPs in solution (0.5 mg/mL) was measured using a Zetasizer Nano Series dynamic light scattering (DLS) instrument (Malvern Instruments). Samples were dissolved in water and incubated at 4 °C for 1 h. Subsequently, they were filtered using a 0.2 μm syringe filter prior to analysis. Measurements were performed in plastic polystyrene cuvettes (BrandTech Scientific) at 25 °C. The laser power was adjusted automatically by the built-in autoattenuation capability for each sample to an optimized range of counts. The acquisition time for each data point was 10 s, and 5 measurements were performed for each sample. The theoretical hydrodynamic diameter was estimated using the formula *D*_*h*_^*IDP*^ = 4.66*N*^0.549^, where *N* is the number of amino acids for each SELP (432 for SE_AI_ and 468 for bSE_AI_).^60^

#### Differential Scanning Calorimetry (DSC)

DSC experiments were carried out in a Mettler Toledo DSC1 device using hermetically sealed aluminum pans. For each sample, a volume of 40 μL of SELP solution (15 wt %) was used. Samples were dissolved in water and incubated at 4 °C for 1 h. Subsequently, samples were subjected to 3 temperature cycles (30 min isotherm at 4 °C, heating ramp from for to 50 °C with a heating rate of 1 °C/min, 30 min isotherm at 50 °C and a cooling ramp of 1 °C/min down to 4 °C).

#### Small-Angle X-ray Scattering (SAXS) and Wide-Angle X-ray Scattering (WAXS)

SAXS and WAXS experiments were performed using a SAXSLAB Ganesha 300XL (Xenocs) microfocus X-ray tube with copper radiation with a motorized collimation system. Dry SELP samples were mounted on tape holders and placed in an evacuated chamber at 0.08 mbar. The scattered intensity was collected by a solid-state photon-counting Pilatus 300 K (Dectris, Switzerland) detector. The sample–detector distance was set to 1041 mm for SAXS measurements and 101 mm for WAXS measurements. The acquisition time was 1800 s for SAXS measurements and 300 s for WAXS measurements. Background signals from empty tape holders were subtracted from each measurement before data analysis. The obtained 2D images were background-corrected, azimuthally averaged, and plotted as 1D scattering profiles using SAXSGUI software.

#### Atomic Force Microscopy (AFM)

Samples for AFM imaging were prepared by diluting dry SELPs in cold Milli-Q water to a final concentration of 0.1 μg/mL. Thereafter, 5 μL of solution were deposited onto a freshly cleaved mica. The sample was left to dry overnight at 4 °C to reduce the thermal motion of SELPs and thus prevent protein aggregation due to rapid evaporation of the aqueous solvent. Samples were subsequently imaged in air using an Icon AFM (Bruker) with ScanAsyst-Air-HPI tips. The AFM was operated using the ScanAsyst Tapping mode at room conditions. The scan area was set to 1 × 1 μm^2^ or 5 × 5 μm^2^, and images were recorded at 512 × 512 pixels and a line rate of 1 Hz. A second-order flattening was performed on all images using NanoScope Analysis 10.0 software.

#### Scanning Electron Microscopy (SEM)

Dry SELPs in different formulations were analyzed by SEM to investigate their microstructure. Samples were coated with a layer of Au/Pd using a SC7620 mini Sputter Coater/Glow Discharge System (Quorum Technologies). SEM images were obtained using a Zeiss GeminiSEM 360 microscope with in-lens detector.

### Computational Simulations

Molecular dynamics (MD) simulations were used to study the interaction between SELPs at the atomistic level. Input structures for MD simulations were created with the SELP sequences shown in Table 1. Extended conformations of each SELP were built using the software Avogadro (version 1.2.0).^61^ MD simulations were performed in Gromacs (2021.2 version)^62^ using the CHARMM36m force field.^63^ The atomic structures were visualized using the Visual Molecular Dynamics (VMD) graphics software.^64^

#### Implicit Solvent Simulations

Each extended SELP structure was subjected to MD simulations in implicit solvent to obtain a folded structure. The equilibration and folding of SELPs was assessed by the evolution of the root mean squared displacement (RMSD) of their atomic positions. First, the structure was subjected to energy minimization for 50,000 timesteps to relax the polypeptide, using the steepest descent algorithm. This was followed by an implicit solvent run at 400 K using a simulation step of 1 fs for a total simulation time of 50 ns. The aim of these simulations was to speed up the folding of the SELP molecules into compact structures to be used in subsequent explicit solvent simulations.

#### Explicit Solvent Simulations

Two copies of the final structures obtained in implicit solvent simulations were solvated in a TIP3P water box with 3D periodic boundary conditions to a final concentration of ca. 150 mg/mL. For bSE_AI_, the charge of the biomineralizing block was neutralized by adding the required NaCl ions. The LINC algorithm was applied to all the bonds containing hydrogen atoms. The resulting systems were equilibrated by subjecting them to energy minimization for 50,000 timesteps, 100 ps using the NVT ensemble at 310 K using a Nosé–Hoover temperature coupling, and 100 ps using the NPT ensemble at 310 K with Parrinello–Rahman pressure coupling. The resulting equilibrated systems were subjected to replica exchange molecular dynamics (REMD). 80 replicas in explicit solvent were used, with temperatures exponentially distributed from 280 to 380 K. A time step of 2 fs was applied, and each replica was simulated for 50 ns, with exchange attempts every 2 ps. The long-range electrostatic Coulombic interactions were calculated using particle mesh Ewald method with a grid spacing of 1.6 Å. A cutoff distance of 10 Å was applied for electrostatic and van der Waals interactions.

#### Data Analysis

A low temperature (7 °C) and a high temperature (37 °C) replicas were subjected to further analysis, to understand the differences in the dynamics of the interaction between SE_AI_ and bSE_AI_. Molecular properties were sampled for the last 10% of the MD trajectories. The package MDAnalysis^65,66^ was used to analyze the number of intermolecular contacts, the intra/interprotein and protein–water hydrogen bonding patterns, the RMSD of the atomic positions, and the radial distribution function. The solvent accessible surface area (SASA), secondary structure, interaction energies and number of water molecules in the solvation shell were calculated using the GROMACS tools gmx sasa, gmx dssp, gmx energy and gmx select, respectively. The distance for the first valley of the RDFs was used as a cutoff to compute the solvation shell (Figure S13).

## Supporting Information Available

Additional NMR spectra, SDS PAGE gels, additional rheological characterization, full FTIR spectra, AFM images of different SELP samples at different magnifications, SAXS/WAXS 2D patterns, LC-HRMS chromatograms and radial distribution function of water molecules in MD simulations (PDF)

## References

[ref1] Harrington M. J.; Fratzl P. Natural load-bearing protein materials. Prog. Mater. Sci. 2021, 120, 100767 10.1016/j.pmatsci.2020.100767.

[ref2] Miserez A.; Yu J.; Mohammadi P. Protein-Based Biological Materials: Molecular Design and Artificial Production. Chem. Rev. 2023, 123 (5), 2049–2111. 10.1021/acs.chemrev.2c00621. 36692900 PMC9999432

[ref3] Harrington M. J.; Mezzenga R.; Miserez A. Fluid protein condensates for bio-inspired applications. Nature Reviews Bioengineering 2024, 2 (3), 260–278. 10.1038/s44222-023-00133-6.

[ref4] Huang W.; Ling S.; Li C.; Omenetto F. G.; Kaplan D. L. Silkworm silk-based materials and devices generated using bio-nanotechnology. Chem. Soc. Rev. 2018, 47 (17), 6486–6504. 10.1039/C8CS00187A. 29938722 PMC6113080

[ref5] Marelli B.; Brenckle M. A.; Kaplan D. L.; Omenetto F. G. Silk Fibroin as Edible Coating for Perishable Food Preservation. Sci. Rep. 2016, 6 (1), 25263 10.1038/srep25263. 27151492 PMC4858704

[ref6] Matzeu G.; Mogas-Soldevila L.; Li W.; Naidu A.; Turner T. H.; Gu R.; Blumeris P. R.; Song P.; Pascal D. G.; Guidetti G.; et al. Large-Scale Patterning of Reactive Surfaces for Wearable and Environmentally Deployable Sensors. Adv. Mater. 2020, 32 (28), 2001258 10.1002/adma.202001258. 32462737

[ref7] Lee A.; Hudson A. R.; Shiwarski D. J.; Tashman J. W.; Hinton T. J.; Yerneni S.; Bliley J. M.; Campbell P. G.; Feinberg A. W. 3D bioprinting of collagen to rebuild components of the human heart. Science 2019, 365 (6452), 482–487. 10.1126/science.aav9051. 31371612

[ref8] Ling S.; Qin Z.; Huang W.; Cao S.; Kaplan D. L.; Buehler M. J. Design and function of biomimetic multilayer water purification membranes. Science Advances 2017, 3 (4), e1601939 10.1126/sciadv.1601939. 28435877 PMC5381955

[ref9] Liu Y.; Gilchrist A. E.; Heilshorn S. C. Engineered Protein Hydrogels as Biomimetic Cellular Scaffolds. Adv. Mater. 2024, 36 (45), 2407794 10.1002/adma.202407794. PMC11573243 39233559

[ref10] Yang Y. J.; Holmberg A. L.; Olsen B. D. Artificially Engineered Protein Polymers. Annu. Rev. Chem. Biomol. Eng. 2017, 8 (1), 549–575. 10.1146/annurev-chembioeng-060816-101620. 28592178

[ref11] El Maachi I.; Loewen A.; Acosta S.; Rütten S.; Rodríguez-Cabello J. C.; Jockenhoevel S.; Fernández-Colino A. Protein-Engineered Elastin Fibers as Building Blocks for The Textile-Based Assembly of Tissue Equivalents. Adv. Funct. Mater. 2024, 34 (24), 2313204 10.1002/adfm.202313204.

[ref12] Urry D. W.; Long M. M.; Cox B. A.; Ohnishi T.; Mitchell L. W.; Jacobs M. The synthetic polypentapeptide of elastin coacervates and forms filamentous aggregates. Biochimica et Biophysica Acta (BBA) - Protein Structure 1974, 371 (2), 597–602. 10.1016/0005-2795(74)90057-9. 4474024

[ref13] López Barreiro D.; Minten I. J.; Thies J. C.; Sagt C. M. J. Structure–Property Relationships of Elastin-like Polypeptides: A Review of Experimental and Computational Studies. ACS Biomaterials Science & Engineering 2023, 9 (7), 3796–3809. 10.1021/acsbiomaterials.1c00145. 34251181

[ref14] Rodríguez-Cabello J. C.; Girotti A.; Ribeiro A.; Arias F. J. Synthesis of Genetically Engineered Protein Polymers (Recombinamers) as an Example of Advanced Self-Assembled Smart Materials. In Nanotechnology in Regenerative Medicine: Methods and Protocols, Navarro M.; Planell J. A., Eds.; Humana Press: 2012; pp 17–38. 10.1007/978-1-61779-388-2_2 22042670

[ref15] Ling S.; Li C.; Adamcik J.; Shao Z.; Chen X.; Mezzenga R. Modulating Materials by Orthogonally Oriented β-Strands: Composites of Amyloid and Silk Fibroin Fibrils. Adv. Mater. 2014, 26 (26), 4569–4574. 10.1002/adma.201400730. 24845975

[ref16] Huang W.; Tarakanova A.; Dinjaski N.; Wang Q.; Xia X.; Chen Y.; Wong J. Y.; Buehler M. J.; Kaplan D. L. Design of Multistimuli Responsive Hydrogels Using Integrated Modeling and Genetically Engineered Silk–Elastin-Like Proteins. Adv. Funct. Mater. 2016, 26 (23), 4113–4123. 10.1002/adfm.201600236. 28670244 PMC5488272

[ref17] Qiu W.; Huang Y.; Teng W.; Cohn C. M.; Cappello J.; Wu X. Complete Recombinant Silk-Elastinlike Protein-Based Tissue Scaffold. Biomacromolecules 2010, 11 (12), 3219–3227. 10.1021/bm100469w. 21058633 PMC3006068

[ref18] Gustafson J. A.; Price R. A.; Greish K.; Cappello J.; Ghandehari H. Silk-Elastin-like Hydrogel Improves the Safety of Adenovirus-Mediated Gene-Directed Enzyme–Prodrug Therapy. Mol. Pharmaceutics 2010, 7 (4), 1050–1056. 10.1021/mp100161u. PMC2933176 20586469

[ref19] Isaacson K. J.; Jensen M. M.; Watanabe A. H.; Green B. E.; Correa M. A.; Cappello J.; Ghandehari H. Self-Assembly of Thermoresponsive Recombinant Silk-Elastinlike Nanogels. Macromol. Biosci. 2018, 18 (1), 1700192 10.1002/mabi.201700192. PMC5806626 28869362

[ref20] Wang E.; Desai M. S.; Lee S.-W. Light-Controlled Graphene-Elastin Composite Hydrogel Actuators. Nano Lett. 2013, 13 (6), 2826–2830. 10.1021/nl401088b. 23647361 PMC3737518

[ref21] Parker R. N.; Wu W. A.; McKay T. B.; Xu Q.; Kaplan D. L. Design of Silk-Elastin-Like Protein Nanoparticle Systems with Mucoadhesive Properties. Journal of Functional Biomaterials 2019, 10 (4), 49 10.3390/jfb10040049. 31726786 PMC6963467

[ref22] Huang W.; Rollett A.; Kaplan D. L. Silk-elastin-like protein biomaterials for the controlled delivery of therapeutics. Expert Opinion on Drug Delivery 2015, 12 (5), 779–791. 10.1517/17425247.2015.989830. 25476201 PMC4579323

[ref23] Cipriani F.; Krüger M.; de Torre I. G.; Sierra L. Q.; Rodrigo M. A.; Kock L.; Rodriguez-Cabello J. C. Cartilage Regeneration in Preannealed Silk Elastin-Like Co-Recombinamers Injectable Hydrogel Embedded with Mature Chondrocytes in an Ex Vivo Culture Platform. Biomacromolecules 2018, 19 (11), 4333–4347. 10.1021/acs.biomac.8b01211. 30346149

[ref24] Ibáñez-Fonseca A.; Orbanic D.; Arias F. J.; Alonso M.; Zeugolis D. I.; Rodríguez-Cabello J. C. Influence of the Thermodynamic and Kinetic Control of Self-Assembly on the Microstructure Evolution of Silk-Elastin-Like Recombinamer Hydrogels. Small 2020, 16 (28), 2001244 10.1002/smll.202001244. 32519515

[ref25] López Barreiro D.; Folch-Fortuny A.; Muntz I.; Thies J. C.; Sagt C. M. J.; Koenderink G. H. Sequence Control of the Self-Assembly of Elastin-Like Polypeptides into Hydrogels with Bespoke Viscoelastic and Structural Properties. Biomacromolecules 2023, 24 (1), 489–501. 10.1021/acs.biomac.2c01405. 36516874 PMC9832484

[ref26] Fernández-Colino A.; Arias F. J.; Alonso M.; Rodríguez-Cabello J. C. Amphiphilic Elastin-Like Block Co-Recombinamers Containing Leucine Zippers: Cooperative Interplay between Both Domains Results in Injectable and Stable Hydrogels. Biomacromolecules 2015, 16 (10), 3389–3398. 10.1021/acs.biomac.5b01103. 26391850

[ref27] Jung H.; Pena-Francesch A.; Saadat A.; Sebastian A.; Kim D. H.; Hamilton R. F.; Albert I.; Allen B. D.; Demirel M. C. Molecular tandem repeat strategy for elucidating mechanical properties of high-strength proteins. Proc. Natl. Acad. Sci. U. S. A. 2016, 113 (23), 6478–6483. 10.1073/pnas.1521645113. 27222581 PMC4988609

[ref28] Rombouts W. H.; de Kort D. W.; Pham T. T. H.; van Mierlo C. P. M.; Werten M. W. T.; de Wolf F. A.; van der Gucht J. Reversible Temperature-Switching of Hydrogel Stiffness of Coassembled, Silk-Collagen-Like Hydrogels. Biomacromolecules 2015, 16 (8), 2506–2513. 10.1021/acs.biomac.5b00766. 26175077

[ref29] Dzuricky M.; Rogers B. A.; Shahid A.; Cremer P. S.; Chilkoti A. De novo engineering of intracellular condensates using artificial disordered proteins. Nat. Chem. 2020, 12 (9), 814–825. 10.1038/s41557-020-0511-7. 32747754 PMC8281385

[ref30] Li N. K.; Roberts S.; Quiroz F. G.; Chilkoti A.; Yingling Y. G. Sequence Directionality Dramatically Affects LCST Behavior of Elastin-Like Polypeptides. Biomacromolecules 2018, 19 (7), 2496–2505. 10.1021/acs.biomac.8b00099. 29665334

[ref31] Rauscher S.; Pomès R. The liquid structure of elastin. eLife 2017, 6, e26526 10.7554/eLife.26526. 29120326 PMC5703643

[ref32] Glassman M. J.; Olsen B. D. Arrested Phase Separation of Elastin-like Polypeptide Solutions Yields Stiff. Thermoresponsive Gels. Biomacromolecules 2015, 16 (12), 3762–3773. 10.1021/acs.biomac.5b01026. 26545151

[ref33] Segvich S. J.; Smith H. C.; Kohn D. H. The adsorption of preferential binding peptides to apatite-based materials. Biomaterials 2009, 30 (7), 1287–1298. 10.1016/j.biomaterials.2008.11.008. 19095299 PMC2744811

[ref34] Meyer D. E.; Chilkoti A. Purification of recombinant proteins by fusion with thermally-responsive polypeptides. Nat. Biotechnol. 1999, 17 (11), 1112–1115. 10.1038/15100. 10545920

[ref35] https://bmrb.io/histogram/ (accessed 2024 17/05/2024).

[ref36] Wang Y.; Jardetzky O. Probability-based protein secondary structure identification using combined NMR chemical-shift data. Protein Sci. 2002, 11 (4), 852–861. 10.1110/ps.3180102. 11910028 PMC2373532

[ref37] Theillet F.-X.; Kalmar L.; Tompa P.; Han K.-H.; Selenko P.; Dunker A. K.; Daughdrill G. W.; Uversky V. N. The alphabet of intrinsic disorder. Intrinsically Disordered Proteins 2013, 1 (1), e24360 10.4161/idp.24360. 28516008 PMC5424786

[ref38] Sebák F.; Szolomájer J.; Papp N.; Tóth G. K.; Bodor A. Proline cis/trans Isomerization in Intrinsically Disordered Proteins and Peptides. Front. Biosci. (Landmark Ed.) 2023, 28 (6), 127 10.31083/j.fbl2806127. 37395034

[ref39] Hu X.; Shmelev K.; Sun L.; Gil E.-S.; Park S.-H.; Cebe P.; Kaplan D. L. Regulation of Silk Material Structure by Temperature-Controlled Water Vapor Annealing. Biomacromolecules 2011, 12 (5), 1686–1696. 10.1021/bm200062a. 21425769 PMC3090511

[ref40] Chen X.; Knight D. P.; Shao Z.; Vollrath F. Regenerated Bombyx silk solutions studied with rheometry and FTIR. Polymer 2001, 42 (25), 09969–09974. 10.1016/S0032-3861(01)00541-9.

[ref41] Cao Y.; Li H. How Do Chemical Denaturants Affect the Mechanical Folding and Unfolding of Proteins?. J. Mol. Biol. 2008, 375 (1), 316–324. 10.1016/j.jmb.2007.10.024. 18021802

[ref42] Garanger E.; MacEwan S. R.; Sandre O.; Brûlet A.; Bataille L.; Chilkoti A.; Lecommandoux S. Structural Evolution of a Stimulus-Responsive Diblock Polypeptide Micelle by Temperature Tunable Compaction of its Core. Macromolecules 2015, 48 (18), 6617–6627. 10.1021/acs.macromol.5b01371.

[ref43] Gonzalez-Obeso C.; Rodriguez-Cabello J. C.; Kaplan D. L. Fast and reversible crosslinking of a silk elastin-like polymer. Acta Biomaterialia 2022, 141, 14–23. 10.1016/j.actbio.2021.12.031. 34971785 PMC8898266

[ref44] Hu X.; Kaplan D.; Cebe P. Determining Beta-Sheet Crystallinity in Fibrous Proteins by Thermal Analysis and Infrared Spectroscopy. Macromolecules 2006, 39 (18), 6161–6170. 10.1021/ma0610109.

[ref45] Morozova S.; Hitimana E.; Dhakal S.; Wilcox K. G.; Estrin D. Scattering methods for determining structure and dynamics of polymer gels. J. Appl. Phys. 2021, 129 (7), 071101 10.1063/5.0033414.

[ref46] Cao Y.; Olsen B. D. Strengthening and Toughening of Protein-Based Thermosets via Intermolecular Self-Assembly. Biomacromolecules 2022, 23 (8), 3286–3295. 10.1021/acs.biomac.2c00372. 35834611

[ref47] Yazawa K.; Ishida K.; Masunaga H.; Hikima T.; Numata K. Influence of Water Content on the β-Sheet Formation, Thermal Stability, Water Removal, and Mechanical Properties of Silk Materials. Biomacromolecules 2016, 17 (3), 1057–1066. 10.1021/acs.biomac.5b01685. 26835719

[ref48] Sun H.; Marelli B. Polypeptide templating for designer hierarchical materials. Nat. Commun. 2020, 11 (1), 351 10.1038/s41467-019-14257-0. 31953407 PMC6969164

[ref49] Li J.; Zhu Y.; Yu H.; Dai B.; Jun Y.-S.; Zhang F. Microbially Synthesized Polymeric Amyloid Fiber Promotes β-Nanocrystal Formation and Displays Gigapascal Tensile Strength. ACS Nano 2021, 15 (7), 11843–11853. 10.1021/acsnano.1c02944. 34251182

[ref50] Chan W. Y.; Bochenski T.; Schmidt J. E.; Olsen B. D. Peptide Domains as Reinforcement in Protein-Based Elastomers. ACS Sustainable Chem. Eng. 2017, 5 (10), 8568–8578. 10.1021/acssuschemeng.7b00698.

[ref51] Mithieux S. M.; Aghaei-Ghareh-Bolagh B.; Yan L.; Kuppan K. V.; Wang Y.; Garces-Suarez F.; Li Z.; Maitz P. K.; Carter E. A.; Limantoro C.; et al. Tropoelastin Implants That Accelerate Wound Repair. Adv. Healthcare Mater. 2018, 7 (10), 1701206 10.1002/adhm.201701206. 29450975

[ref52] Xiao Y.; Liu Y.; Zhang W.; Qi P.; Ren J.; Pei Y.; Ling S. Formation, Structure, and Mechanical Performance of Silk Nanofibrils Produced by Heat-Induced Self-Assembly. Macromol. Rapid Commun. 2021, 42 (3), 2000435 10.1002/marc.202000435. 33196127

[ref53] Nguyen A. T.; Huang Q.-L.; Yang Z.; Lin N.; Xu G.; Liu X. Y. Crystal Networks in Silk Fibrous Materials: From Hierarchical Structure to Ultra Performance. Small 2015, 11 (9–10), 1039–1054. 10.1002/smll.201402985. 25510895

[ref54] Adamcik J.; Mezzenga R. Amyloid Polymorphism in the Protein Folding and Aggregation Energy Landscape. Angew. Chem., Int. Ed. 2018, 57 (28), 8370–8382. 10.1002/anie.201713416. 29446868

[ref55] Mu X.; Yuen J. S. K.; Choi J.; Zhang Y.; Cebe P.; Jiang X.; Zhang Y. S.; Kaplan D. L. Conformation-driven strategy for resilient and functional protein materials. Proc. Natl. Acad. Sci. U. S. A. 2022, 119 (4), e2115523119 10.1073/pnas.2115523119. 35074913 PMC8795527

[ref56] Kamada A.; Rodriguez-Garcia M.; Ruggeri F. S.; Shen Y.; Levin A.; Knowles T. P. J. Controlled self-assembly of plant proteins into high-performance multifunctional nanostructured films. Nat. Commun. 2021, 12 (1), 3529 10.1038/s41467-021-23813-6. 34112802 PMC8192951

[ref57] Pena-Francesch A.; Jung H.; Demirel M. C.; Sitti M. Biosynthetic self-healing materials for soft machines. Nat. Mater. 2020, 19 (11), 1230–1235. 10.1038/s41563-020-0736-2. 32719508 PMC7610468

[ref58] Varanko A. K.; Su J. C.; Chilkoti A. Elastin-Like Polypeptides for Biomedical Applications. Annu. Rev. Biomed. Eng. 2020, 22 (1), 343–369. 10.1146/annurev-bioeng-092419-061127. 32343908

[ref59] Vente A.; Mladic M.; López Barreiro D.; Schouten O.; van der Hoeven R. Characterization of elastin-like polypeptides combining a novel proalanase bottom-up approach and intact protein analysis; IMSC: Maastricht, the Netherlands, 2022.

[ref60] Marsh J. A.; Forman-Kay J. D. Sequence Determinants of Compaction in Intrinsically Disordered Proteins. Biophys. J. 2010, 98 (10), 2383–2390. 10.1016/j.bpj.2010.02.006. 20483348 PMC2872267

[ref61] Hanwell M. D.; Curtis D. E.; Lonie D. C.; Vandermeersch T.; Zurek E.; Hutchison G. R. Avogadro: an advanced semantic chemical editor, visualization, and analysis platform. Journal of Cheminformatics 2012, 4 (1), 17 10.1186/1758-2946-4-17. 22889332 PMC3542060

[ref62] Abraham M. J.; Murtola T.; Schulz R.; Páll S.; Smith J. C.; Hess B.; Lindahl E. GROMACS: High performance molecular simulations through multi-level parallelism from laptops to supercomputers. SoftwareX 2015, 1–2, 19–25. 10.1016/j.softx.2015.06.001.

[ref63] Huang J.; Rauscher S.; Nawrocki G.; Ran T.; Feig M.; de Groot B. L.; Grubmüller H.; MacKerell A. D. CHARMM36m: an improved force field for folded and intrinsically disordered proteins. Nat. Methods 2017, 14 (1), 71–73. 10.1038/nmeth.4067. 27819658 PMC5199616

[ref64] Humphrey W.; Dalke A.; Schulten K. VMD: Visual molecular dynamics. J. Mol. Graphics 1996, 14 (1), 33–38. 10.1016/0263-7855(96)00018-5. 8744570

[ref65] Michaud-Agrawal N.; Denning E. J.; Woolf T. B.; Beckstein O. MDAnalysis: A toolkit for the analysis of molecular dynamics simulations. J. Comput. Chem. 2011, 32 (10), 2319–2327. 10.1002/jcc.21787. 21500218 PMC3144279

[ref66] Gowers R. J.; Linke M.; Barnoud J.; Reddy T. J. E.; Melo M. N.; Seyler S. L.; Domański J.; Dotson D. L.; Buchoux S.; Kenney I. M.; Beckstein O. MDAnalysis: A Python Package for the Rapid Analysis of Molecular Dynamics Simulations. In Proceedings of the Python in Science Conference; Benthall S.; Rostrup S., Eds.; 2016; pp 98-105. DOI: https://www.doi.org/10.25080/Majora-629e541a-00e.

