# Supplementary material for: Order–Disorder Balance in Silk-Elastin-like Polypeptides Determines Their Self-Assembly into Hydrogel Networks
Source: ACS Appl Mater Interfaces. 2024 Dec 16;17(1):650–62. doi: 10.1021/acsami.4c17903 (PMC11783522; doi:10.1021/acsami.4c17903)
Supplement: Supplementary file 1 — am4c17903_si_001.pdf [file am4c17903_si_001.pdf]

SUPPORTING INFORMATION

Order-disorder balance in silk-elastin-like polypeptides determines their self-assembly into hydrogel networks

Diego López Barreiro[1,2,3*], Klaartje Houben[4], Olaf Schouten[4],

Gijsje H. Koenderink[5], Jens C. Thies[6], Cees M. J. Sagt[3]

[1]Manufacturing Futures Lab, Department of Chemical Engineering, University College London, London, WC1E 7JE, United Kingdom

[2]Centre for Nature-Inspired Engineering, Department of Chemical Engineering, University College London, London, WC1E 7JE, United Kingdom

[3]dsm-firmenich Science & Research, Biotechnology, Alexander Fleminglaan 1, 2613 AX Delft, The Netherlands

[4]dsm-firmenich Science & Research, Analytical Sciences, Alexander Fleminglaan 1, 2613 AX Delft, The Netherlands

[5]Department of Bionanoscience, Kavli Institute of Nanoscience Delft, Delft University of Technology, Van der Maasweg 9, 2629 HZ Delft, The Netherlands

[6]DSM Biomedical, Urmonderbaan 22, 6160 BB, Geleen, The Netherlands

**Corresponding author: d.lopezbarreiro@ucl.ac.uk*

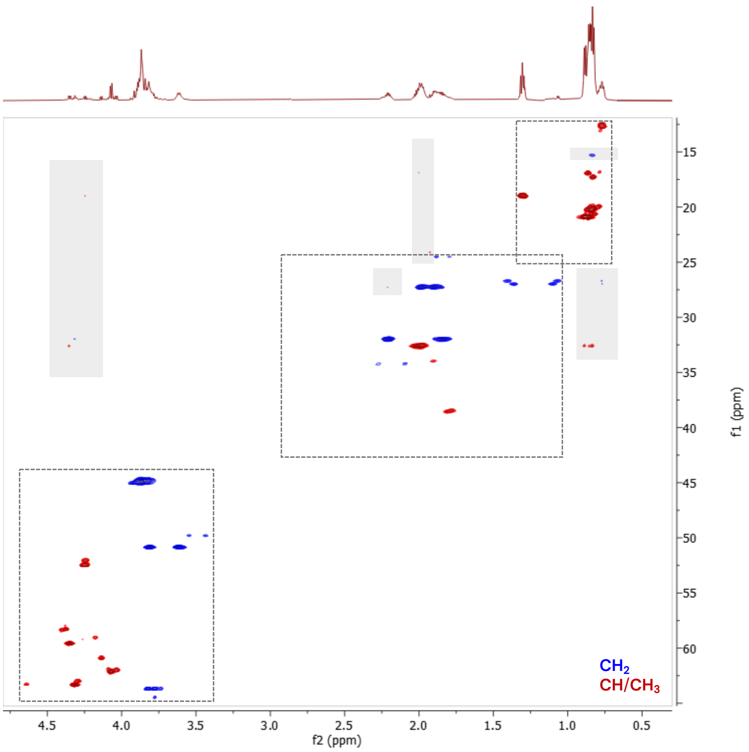

Figure S1 *- 2D edited ^{1}H-^{13}C HSQC spectrum of SE$_{AI}$ shows specific chemical shifts for residues in different regions. CH and CH$_3$ groups give positive (red) signals and CH$_2$ groups negative (blue) signals. Grey areas indicate regions with base-line distortions and blue or red signals observed there are not cross-peaks from the protein.*

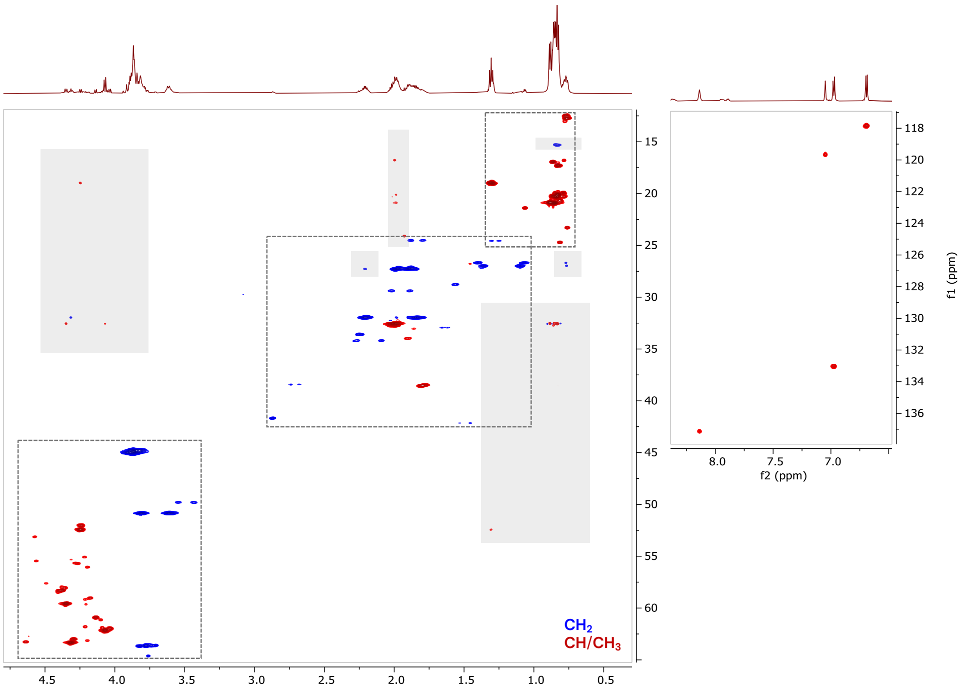

Figure S2 *- 2D edited ^{1}H-^{13}C HSQC spectrum of bSE$_{AI}$ shows specific chemical shifts for residues in different regions. CH and CH$_3$ groups give positive (red) signals and CH$_2$ groups negative (blue) signals. The insert shows the additional region of the spectrum where the aromatic residues H and Y are observed. Grey areas indicate regions with base-line distortions and blue or red signals observed there are not cross-peaks from the protein.*

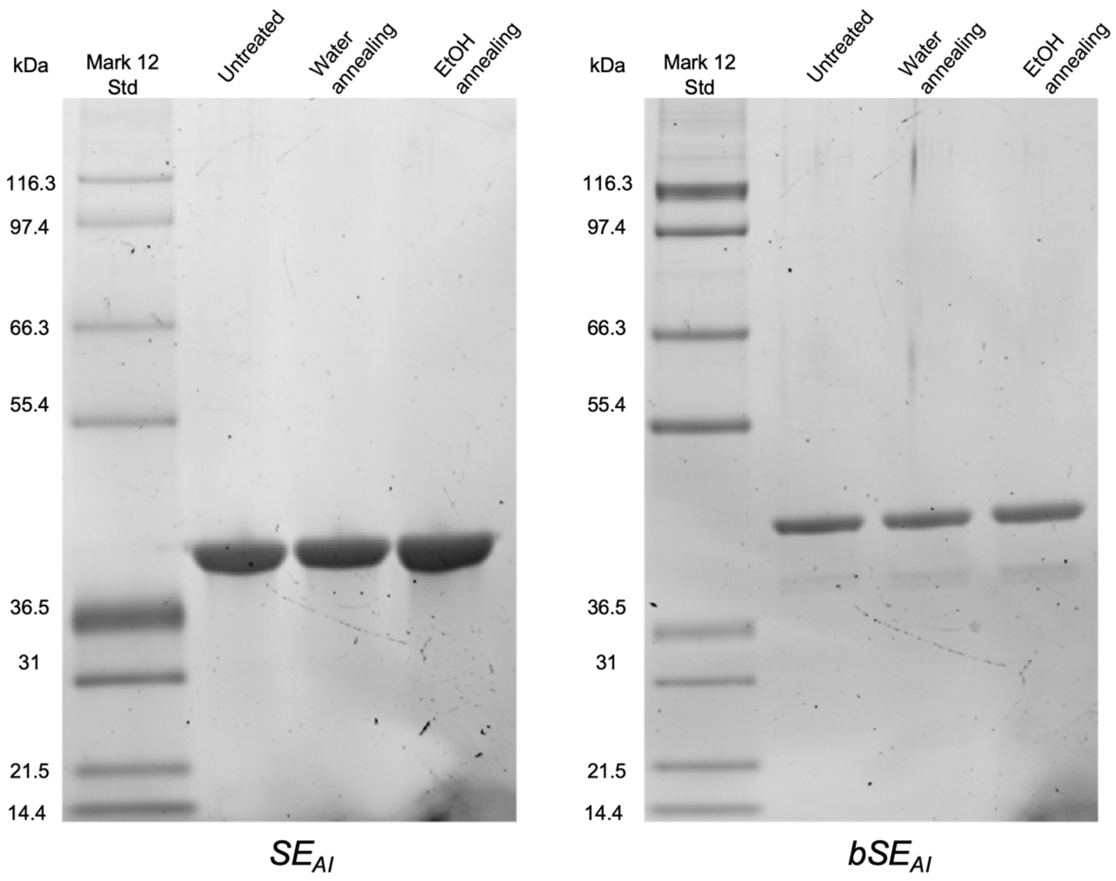

Figure S3 *– SYPRO® Red-stained SDS-PAGE gel of SELPs with different formulations. The left lane is the Mark12 unstained protein MW standard (Thermo Fisher Scientific).*

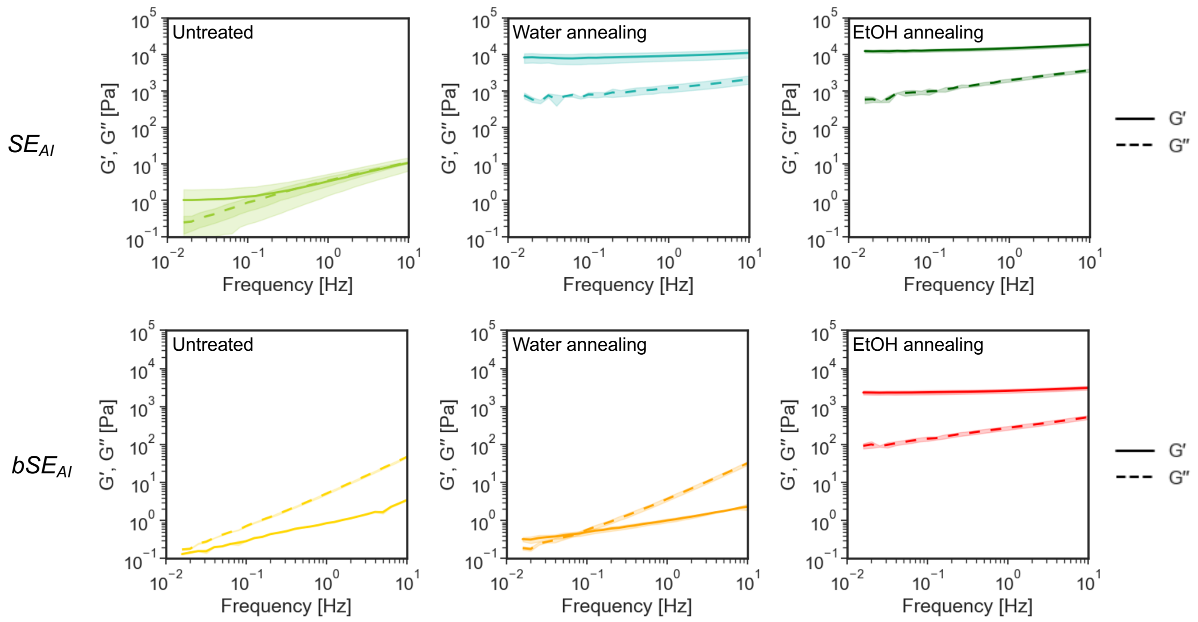

Figure S4 *– Frequency sweeps (γ=0.01-15 %, f=1 Hz) of SELP solutions (15 wt % in milliQ water) (n=2).*

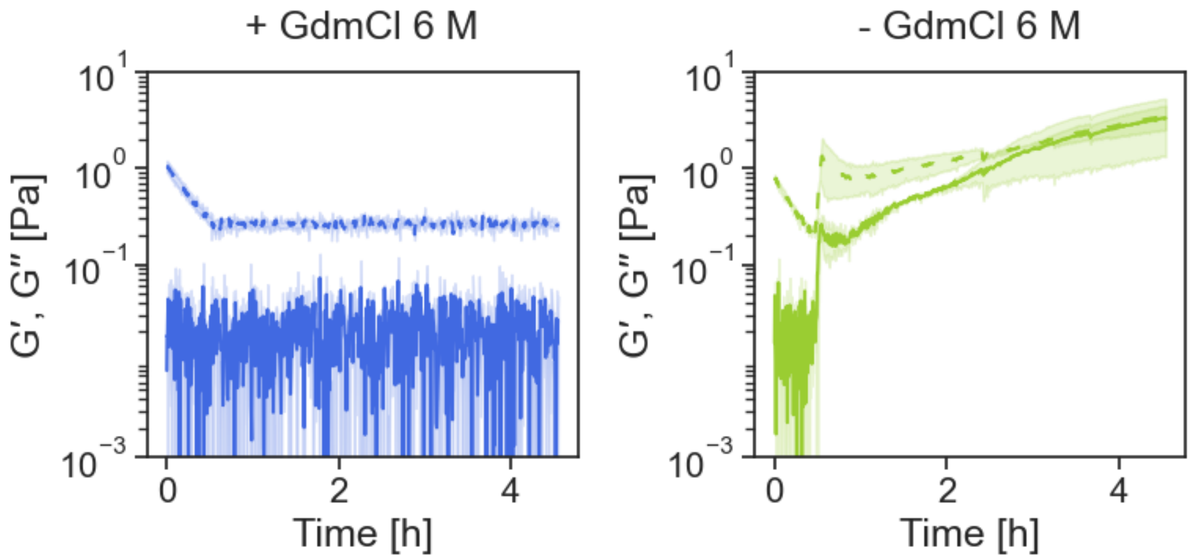

Figure S5 *– Temperature ramp (4-37 °C, 1 °C/min) followed by a time sweep (4 h, 37 °C) for 15 wt % SELP solutions in milliQ water with and without GdmCl 6M, γ=0.3%, f=1 Hz (n=2).*

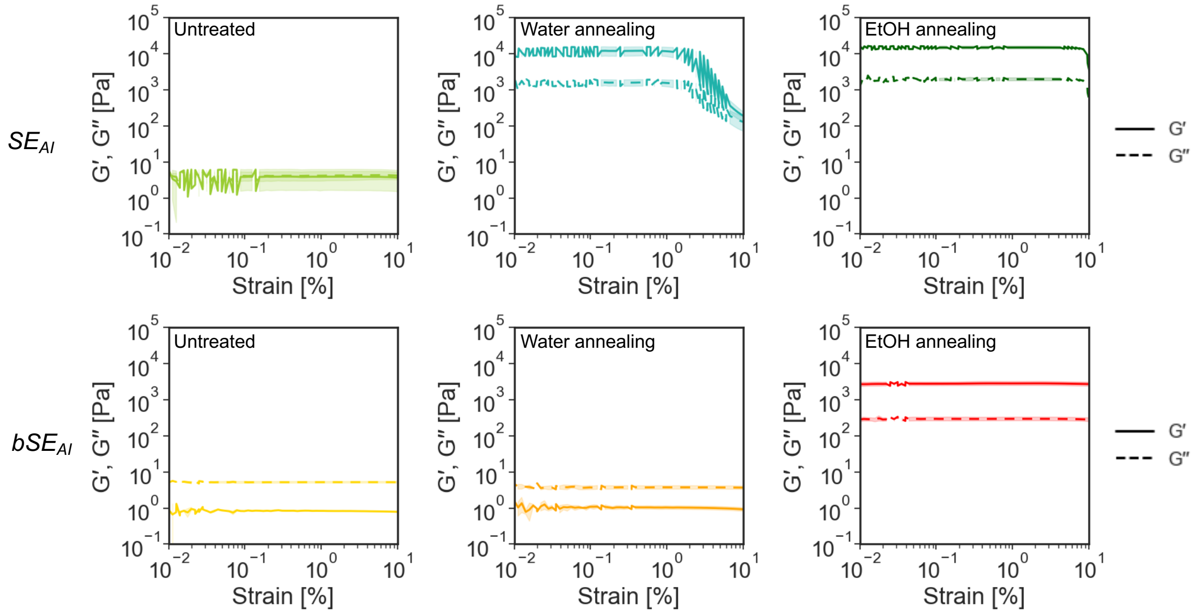

Figure S6 *– Amplitude sweeps (f=0.01-15 Hz, γ=0.3%.) of 15 wt % SELP solutions in milliQ water (n=2).*

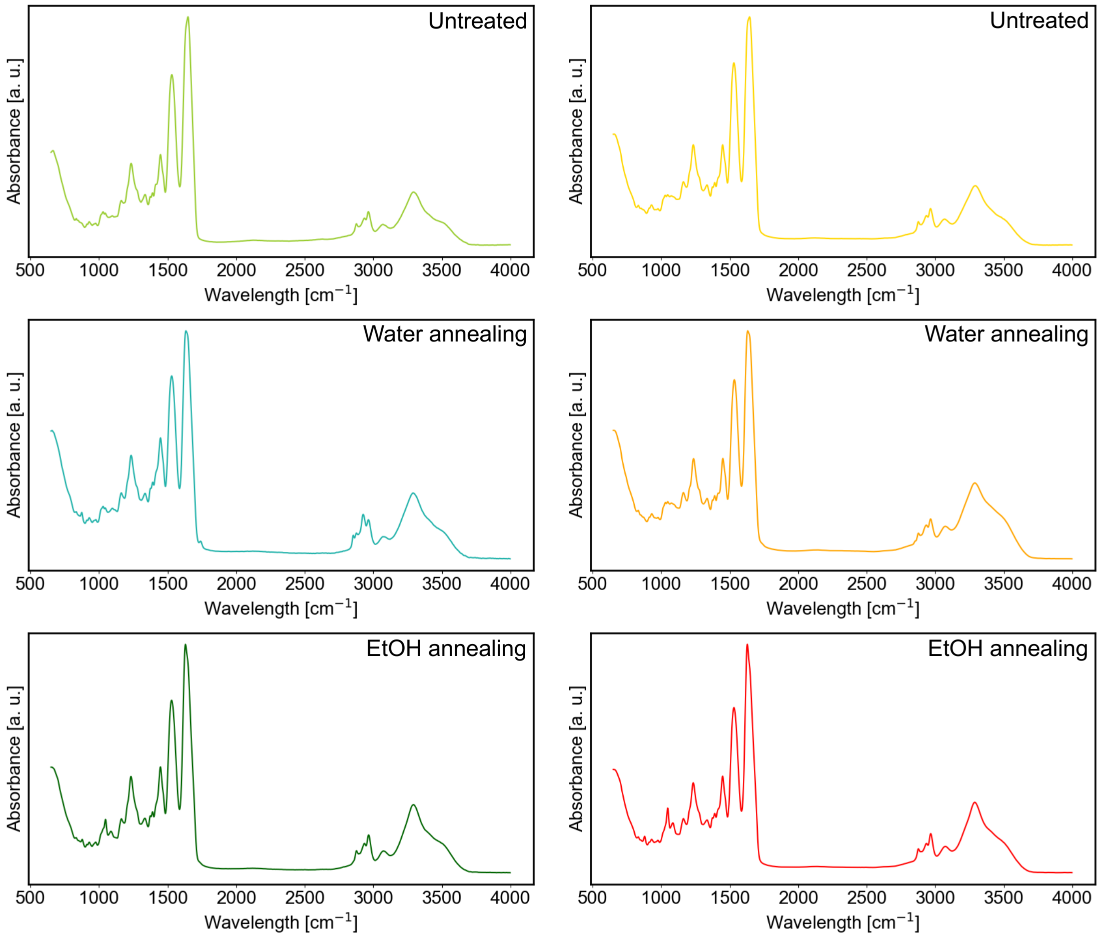

***Figure S7** – Full FTIR spectra for the different SELP formulations tested in this study.*

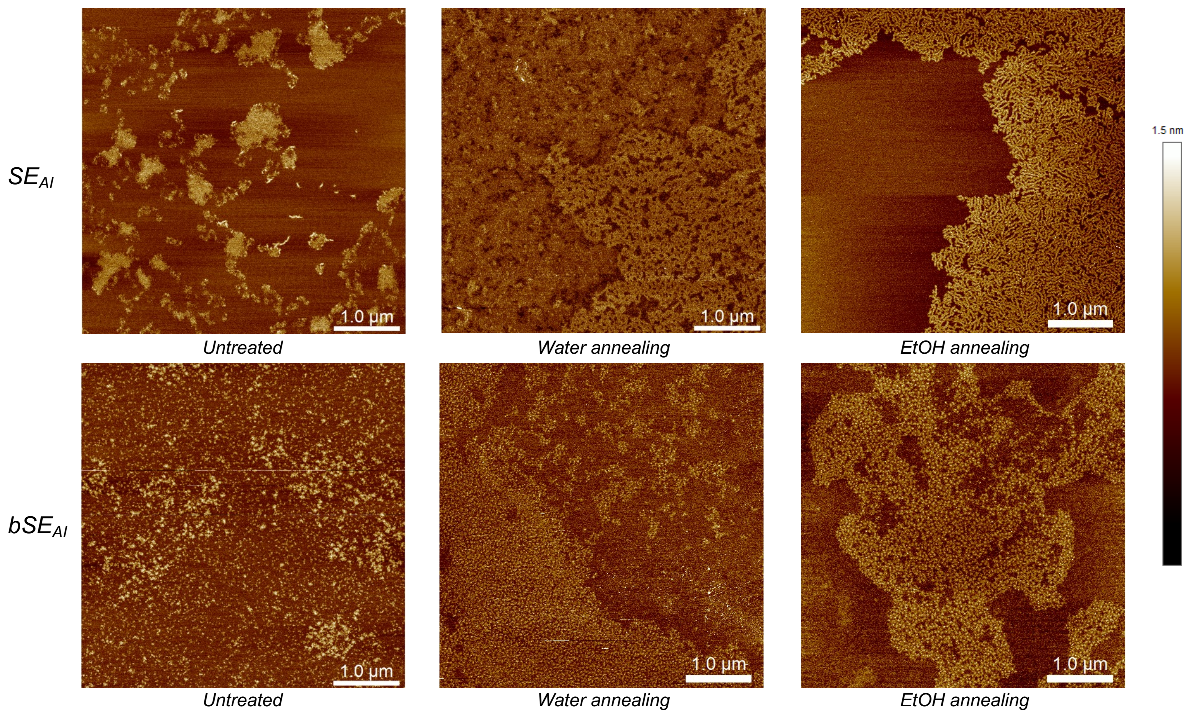

Figure S8 *– AFM images of SELP networks in different formulations.*

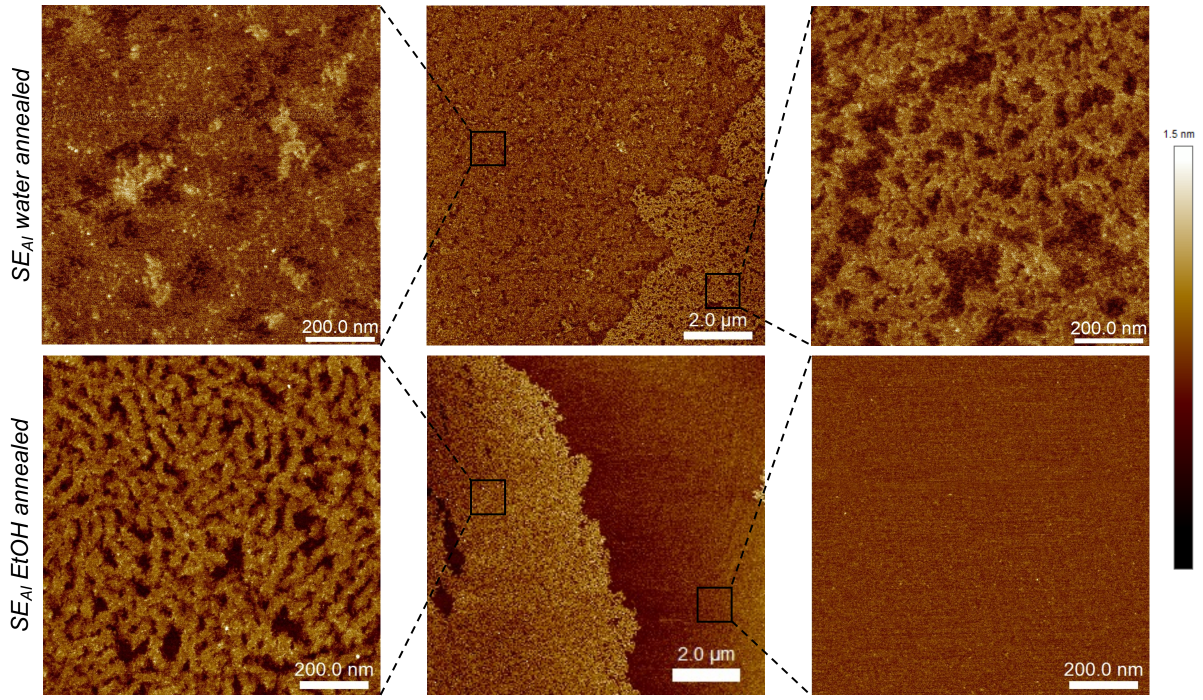

Figure S9 *– AFM images of SE_{AI} networks after water annealing (top) and after EtOH annealing (bottom). Water-annealed SE_{AI} shows a mixture of short and long fibrillar structures, whereas EtOH-annealed SEAI appears to have a more homogeneous composition.*

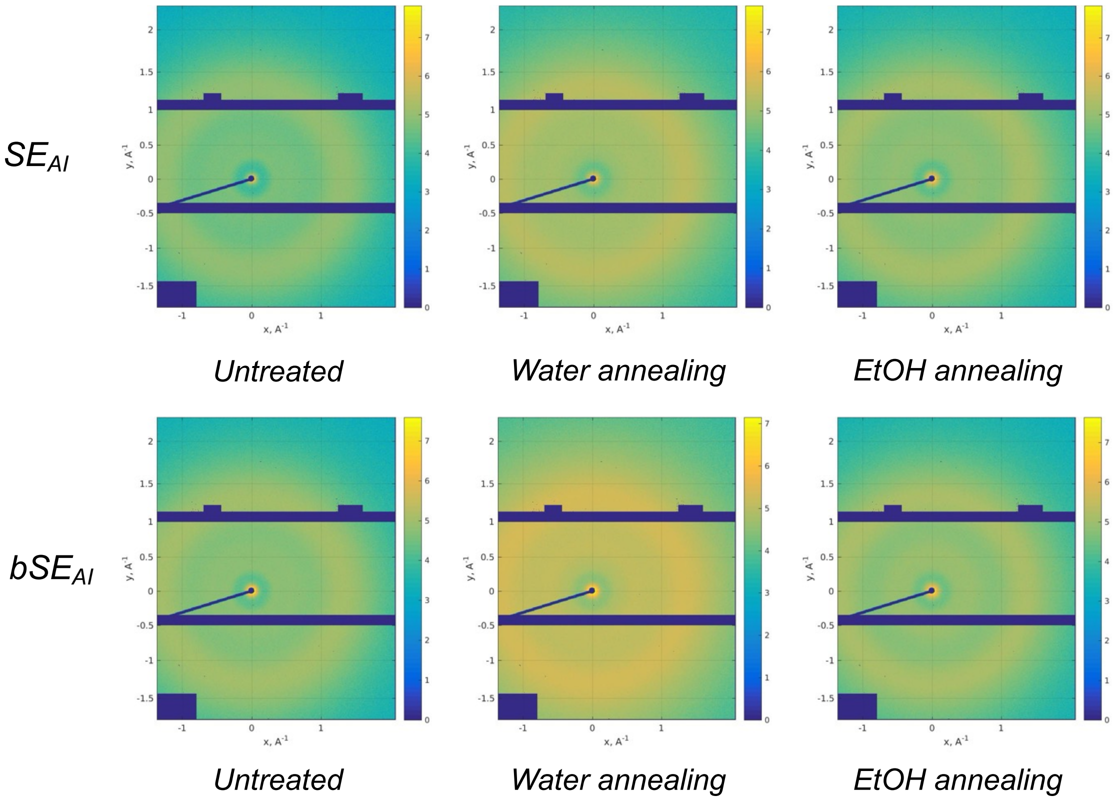

Figure S10 *– 2D WAXS patterns for SELPs in different formulations.*

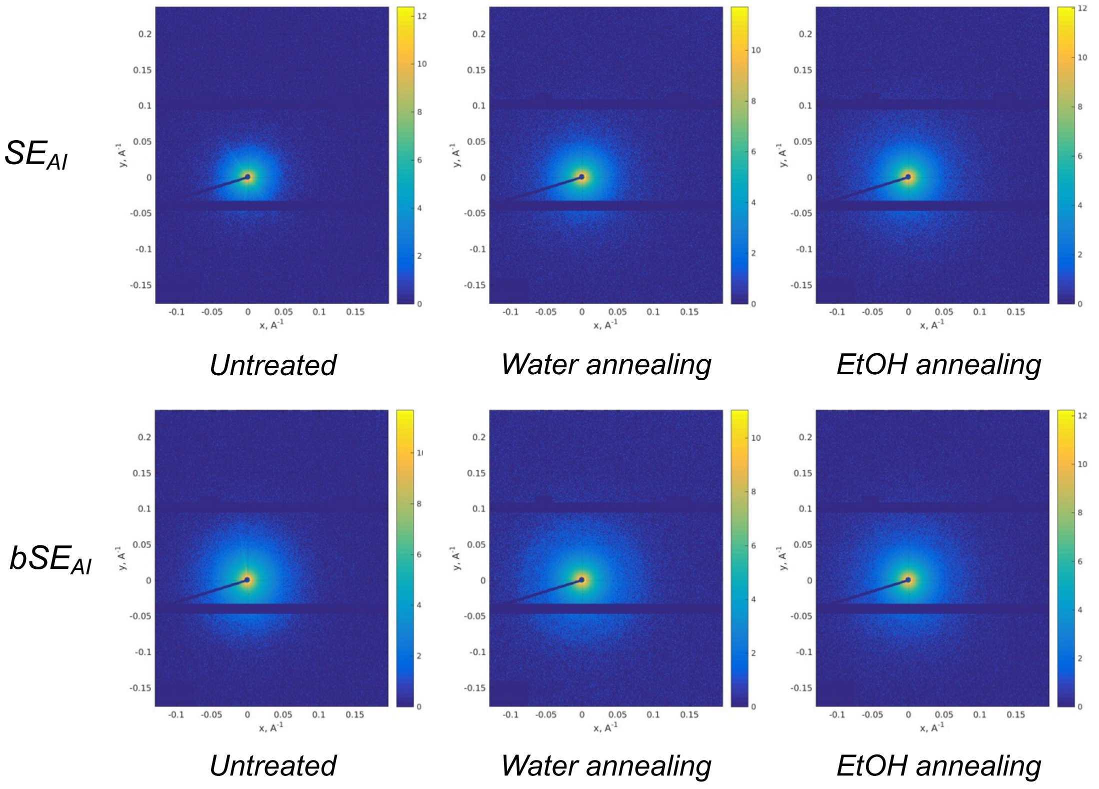

Figure S11 *– 2D SAXS patterns for SELPs in different formulations.*

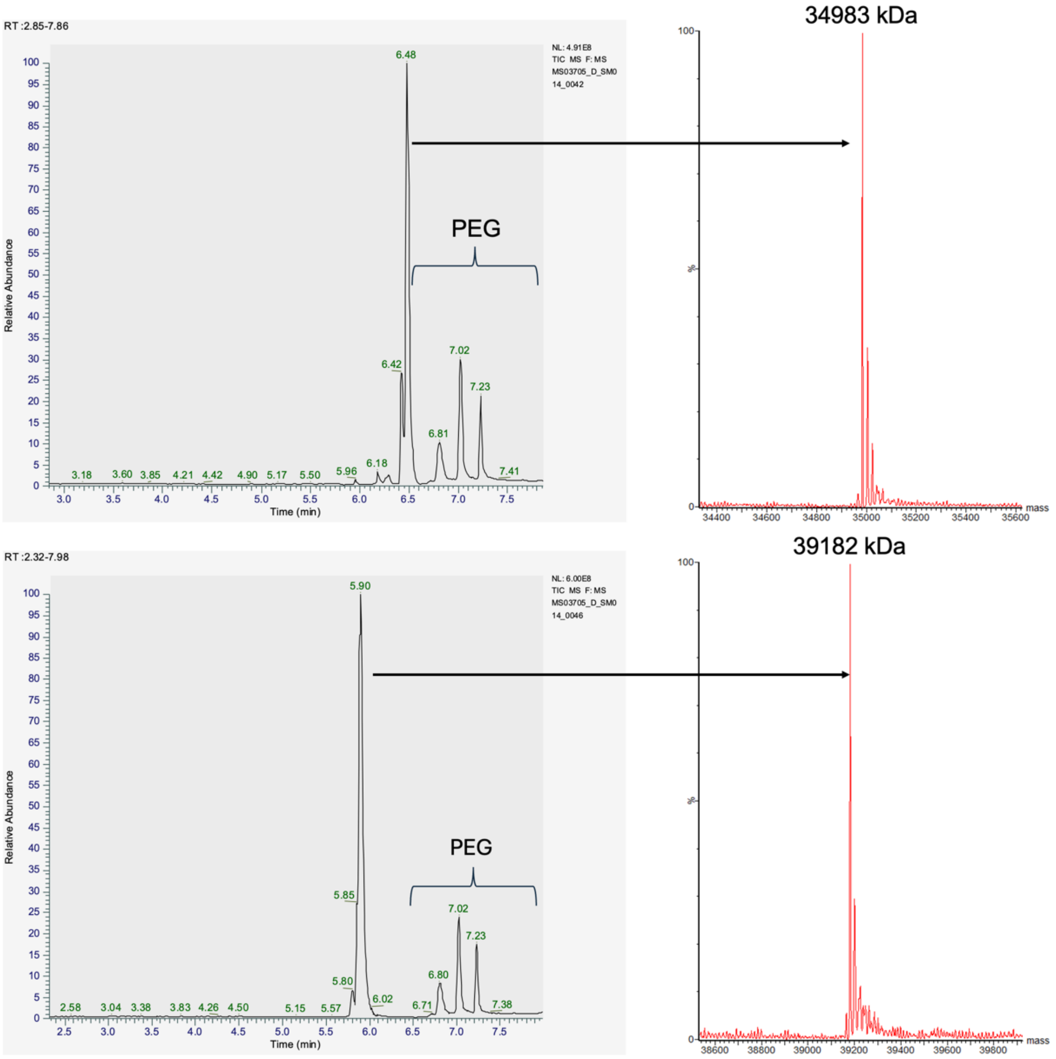

Figure S12 *– LC-HRMS chromatogram (left column) and deconvoluted MS spectrum (right column) for* SE_{AI} *(top) and* bSE_{AI} *(bottom). PEG=polyethylene glycol*

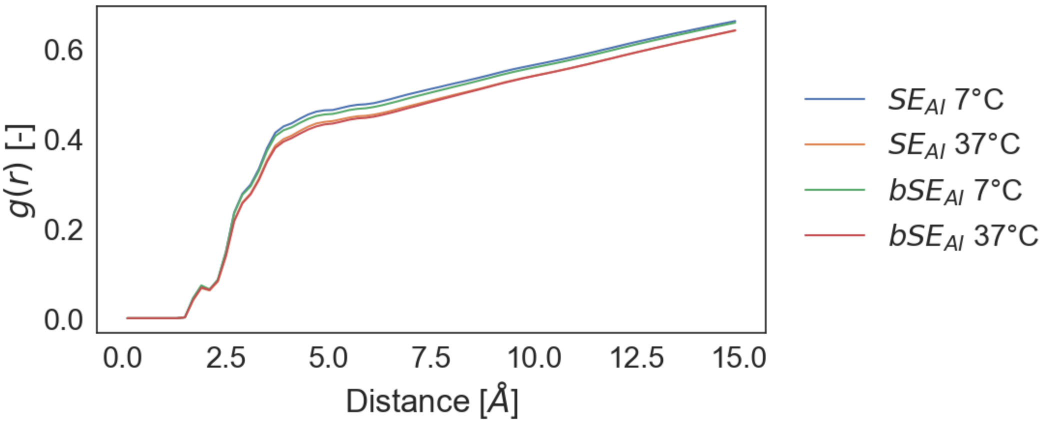

Figure S13 *– Radial distribution function g(r) of water molecules within a certain distance from SELP molecules for REMD simulations. The distance for the first valley of the RDFs was used as a cutoff to compute the solvation shell.*
